# Nationally representative estimates of the cost of adequate diets, nutrient level drivers, and policy options for households in rural Malawi

**DOI:** 10.1016/j.foodpol.2022.102275

**Published:** 2022-11

**Authors:** Kate R. Schneider

**Affiliations:** Johns Hopkins University, Paul H. Nitze School of Advanced International Studies, Washington, DC, USA

**Keywords:** Least-cost diets, Nutrient requirements, Shadow prices, Policy modeling, Linear programming, Nutritious diets, Biofortification

## Abstract

A growing literature uses least-cost diets to evaluate how effectively a food system supports access to nutritious foods. We identify the cost of meeting nutrient requirements for whole households in rural Malawi from and the nutrient-level drivers thereof. From 2013 to 2017, we can identify a household least-cost diet only 60% of the time with an average cost of $2.32/person/day (2011 US$ PPP). We illustrate that larger households have more diverse nutrient needs and face a higher cost for 1000 calories of a sufficiently nutrient dense diet. Shadow price analysis shows riboflavin to be the costliest nutrient in the market. We use policy scenarios to understand what drives the infeasibility and high cost. Simulating the impact of selenium soil biofortification of maize results in a feasible diet 94% of the time at half the cost ($1.22/person/day on average) and eliminates the shadow price of copper. This is explained by insufficient selenium from sources low in copper such that under baseline conditions it is impossible to get enough selenium without too much copper. Even when feasible, to avoid copper, more higher cost foods enter the diet than would be otherwise needed to meet remaining nutrient requirements. Other value chain scenarios to increase the availability and lower the cost of nutrient-dense foods did not meaningfully change the diet cost results. Of direct relevance to Malawi, this study demonstrates more broadly how least-cost diet methods can be used to assess barriers to accessing an adequate diet and the potential impacts of policy options.

## Highlights

• Malawi’s food system is currently unable to reliably facilitate access to nutrient-adequate diets for rural families.
• Larger households eating shared meals face greater constraints.
• Several nutrients drive the observed high cost: riboflavin, B12, copper, and selenium.
• Selenium soil biofortification of maize can make adequate diets near-universally feasible at half the cost.
• Policy scenario and least-cost diet modeling can identify barriers and opportunities to accessing nutritious diets for all.

## 1 Introduction

Good nutrition supports optimal growth and long-term health, yet many food systems are unable to facilitate access to a nutritious diet for all. Least-cost diets offer a food system metric that is flexible to changes in the availability and price of foods, considering all food items that could supply required nutrients while keeping total diet cost at a minimum (Allen, 2017, FAO, 2020, Masters et al., 2018). Most research on nutritionally adequate least-cost diets has focused on a few representative types of persons, since nutrition needs vary by age, sex, physical activity, and reproductive status. Our approach is unique in focusing on the household as the unit that procures food and eats shared meals. It is not only a simple aggregation of members and their individual needs but incorporates the reality that a shared meal must be denser in each nutrient (quantity per unit of energy) to meet the minimum need of the member with the greatest requirement (Schneider et al., 2021b). The household least-cost diets we calculate using subnational retail market prices identify how well national food systems can deliver diets that meet the needs of all members of the population in the proportions required when families share meals.

We run nearly 1.5 million linear models to estimate the least-cost diet and nutrient shadow prices for 3117 households (15,374 individuals) observed in Malawi’s Integrated Household Panel Survey (IHPS). We match each household to the central market in its district where monthly food prices are systematically collected by the National Statistics Office (NSO) for 51 regularly consumed food items in order to calculate the consumer price index (Kaiyatsa et al., 2021). We use linear programming to calculate the monthly least-cost diet meeting the household’s total energy need, satisfying its minimum requirements for 21 nutrients, and not exceeding limits for the 13 nutrients where upper bounds are defined (Schneider et al., 2021b). The dual result to the linear optimization problem calculates the shadow price of each constraint, which demonstrates which nutrients drive the cost on the margin. We model observed data as the base case and eight policy scenarios modifying the availability and/or price of certain items. We select the policy scenarios based on the shadow price results and analysis of current diets, to focus on food sources of the nutrients most under-consumed in present diets and costliest to obtain. The scenarios reflect stylized outcomes of feasible interventions, they help reveal what drives the base case results. If the results are driven by limited availability and high price of these nutrient-dense foods, comparing scenarios will suggest the most effective efforts to improve access to a nutrient-adequate diet.

Most Malawians consume diets that are poor in overall quality, meaning they lack in total quantity and in presence of all food groups in recommended amounts, which leads to risks of micronutrient deficiencies. Current diets are highly imbalanced with the majority of energy from a single food: maize (Gelli et al., 2020, Gilbert et al., 2019). In prior research, we found households to be consuming diets with too many carbohydrates and too few of many other nutrients, especially phosphorus, riboflavin, lipids, selenium, and vitamin B12 (see supplementary materials, Table B-1 for an illustration of adequacy ratios) (Schneider et al., 2021b). This is consistent with findings from primary research on micronutrient status, soils, and food composition, as well as other household survey analyses (Aberman et al., 2018, Gilbert et al., 2019, Hurst et al., 2013, Joy et al., 2015a, Joy et al., 2015b, Phiri et al., 2019). All essential nutrients are necessary for optimal growth and long-term health and deficiencies impede the many body functions to which each contributes directly, as well as the functions played by other nutrients with overlapping roles. For instance, selenium is an important antioxidant and deficiencies in selenium draw down the body’s resources of other nutrients with this same function – e.g. vitamin C, vitamin E, and zinc – reducing the quantities available for the other functions those nutrients perform such as immunity and growth (Byrd-Bredbenner et al., 2016, Institute of Medicine of the National Academies, 2006).

Whether nutritious diets are available and/or affordable is of key concern for policymakers and an active area of research (for a comprehensive discussion, see FAO et al. 2020). Globally, low-income consumers are more likely to have poorer quality diets and health outcomes than wealthier consumers; food prices are often implicated to explain this disparity in countries across the income spectrum (Bai et al., 2021, Darmon and Drewnowski, 2015, Fan et al., 2018, Jones et al., 2018, Vellakkal et al., 2015). The appeal of a least-cost diet approach is that the food basket changes in response to availability and prices and is agnostic to the exact food mix as long as requirements are met (Herforth et al., 2020, Masters et al., 2018). Calculating the diet cost at the household level incorporates the reality that families procure food as a unit and can be compared to incomes, typically also measured at the household level (Schneider et al., 2021a). A household least-cost diet metric is consistent with the information commonly used for food and social protection policy and can be modeled at the same spatial and temporal disaggregation as the underlying food price data (Bai et al., 2021, Dizon et al., 2019, Herforth et al., 2020, Herforth et al., 2019, Masters et al., 2018, Raghunathan et al., 2021, Schneider et al., 2021a). In contrast to traditional consumer price indices, least-cost diets demonstrate the movement of food prices in the proportions corresponding to the nutrient needs of the population.

This paper proceeds as follows. Section 2 summarizes the related literature. Section 3 presents the data, methods, linear optimization model, and policy scenarios. Section 4 presents the results. Section 5 concludes.

## 2 Related literature

Our study is motivated by, and extends, several existing bodies of literature. The first identifies least-cost diets meeting specific nutrition requirements or food-based dietary guidelines to track the cost of those diets (Allen, 2017, Herforth et al., 2019, Masters et al., 2018, O’Brien-Place and Tomek, 1983, Stigler, 1945). Similar applications develop recommended cost-minimizing diets for low-income consumers (Carlson et al., 2007, Chastre et al., 2009, Frega et al., 2012, Wfp, 2013). The second focuses on the relationship between food source (production vs. market) and nutrition (Headey et al., 2019, Hirvonen and Hoddinott, 2017, Hoddinott et al., 2015, Romeo et al., 2016, Sibhatu and Qaim, 2018a, Stifel and Minten, 2017). The third estimates nutrient shadow prices, largely concentrated on the demand side (Beatty, 2007, Drèze and Stern, 1990). We contribute to the small but growing literature using optimization to estimate shadow prices in markets (Darmon et al., 2002, Håkansson, 2015, Parlesak et al., 2016).

### 2.1 Least-cost diets

Least-cost diets have been applied in numerous contexts to track the cost of foods and nutrients, plan diets and menus, and make dietary recommendations for low-income populations (Akhter et al., 2018, Allen, 2017, Calkins, 1981, Carlson et al., 2007, Chastre et al., 2009, Deptford et al., 2017, Masters et al., 2018, Optifood, 2012, Ravallion, 2020, Stigler, 1945, van Dooren, 2018, Wilde, 2013). They are relatively straightforward to compute, requiring data on the available food items, their nutrient composition or food group, market prices, and nutrition requirements (needed amounts of each nutrient or food group). Nutrition constraints can be specified as the quantity of each essential nutrient needed according to international standards or according to other standards of diet quality such as dietary diversity or food-based dietary guidelines (Chastre et al., 2009, Daelmans et al., 2013, Dizon et al., 2019, Herforth et al., 2020, Herforth et al., 2019, Masters et al., 2018, Optifood, 2012, Raghunathan et al., 2021, Schneider et al., 2021a).

Numerous studies have confirmed that nutrient-adequate diets remain out of reach for most of the world’s poorest (Allen, 2017, Bai et al., 2021, Herforth et al., 2020, Schneider et al., 2021a). In India, Raghunathan et al. (2021) showed that the daily cost comprises more than half of daily unskilled wages for men and an even larger share for women. In Uganda, Omiat and Shively (2017) found the cost of a diet meeting minimum nutrient needs increased from 2000 to 2011 and mostly remained above the poverty line. In France, Maillot et al. (2017) found that the lowest income consumers could not feasibly move from an inadequate to adequate diet without increasing total food budgets. In Ghana, Masters et al. (2018) showed the cost of meeting nutrient adequacy doubled from 2009 to 2014 and pronounced seasonal fluctuations in Tanzania, though the total diet cost changed only slightly.

The studies above focus on individuals, while our emphasis is on households where there is only a small preceding literature. Calkins (1981) modeled the optimal crop and livestock mix to maximize both profit and nutrition for a representative Nepalese household, and found shifting towards greater production of potatoes, radishes, rape would be more nutritious and profitable. Chastre et al. (2009) calculated least-cost diets for representative households in Bangladesh, Myanmar, Ethiopia, and Tanzania. We extend this literature by modeling the cost of meeting all nutrient needs for a nationally representative sample of households.

### 2.2 Role of markets in nutrition

The development economics literature has demonstrated a variety of possible linkages between a household or village’s own agricultural production and their food consumption. The literature has shown that production and consumption decisions for most smallholder farming households in low-income countries are linked, but that when households have access to markets they become less so and consumption patterns become more diverse than households’ own agricultural production (de Janvry et al., 1991, Hoddinott et al., 2015). Rural food markets are increasingly recognized as important complements to a household’s own farm production, especially for more perishable and nutrient-dense foods (Headey et al., 2019, Hirvonen and Hoddinott, 2017, Sibhatu and Qaim, 2018a). Studies have found that even smallholder farming households typically rely on purchased food to increase the diversity, stability and quality of diets (Gelli et al., 2020, Headey et al., 2019, Hirvonen et al., 2017, Koppmair et al., 2016, Sibhatu and Qaim, 2018b). Similarly, analyses of programs focused on increasing households’ own production of nutrient-dense foods have sometimes found positive effects on dietary diversity and other measures of nutrition, but also demonstrated a clear mediating role of household access to markets (Fraval et al., 2020, Jones, 2017, Kumar et al., 2015, Lehmann-Uschner and Kraehnert, 2016, Leroy and Frongillo, 2007, Masset et al., 2012, Romeo et al., 2016, Ruel et al., 2018, Sibhatu and Qaim, 2017, Sibhatu and Qaim, 2018a, Sibhatu and Qaim, 2018b, Talukder et al., 2010, van den Bold et al., 2013, Wood et al., 2013). Markets are particularly important complements for households that rely on nonfarm earnings to meet their food needs, but even most smallholder farmers are net buyers of food, including in rural Malawi (Dorward et al., 2004, Headey, 2016, Ivanic and Martin, 2014).

Policymakers and development programs focus on increasing incomes as the long-term, sustainable pathway out of poverty. But the purchasing power of that income depends on the availability and prices of foods at local markets, which may not reliably supply enough nutrients to meet the population’s needs if nutrient-dense foods are not consistently and affordably present. Seasonality, lack of storage opportunities, poor market integration, and limited transport options for perishable items can lead to high and/or volatile food prices and periodic lack of availability. The combined effect of availability and prices may hinder the potential for income growth to improve diet quality and nutrition without additional food systems interventions (Gelli et al., 2020, Herforth and Ahmed, 2015).

### 2.3 Nutrient-level drivers of diet costs

Identifying shadow prices from mathematical programming models of market prices relative to nutritional needs could help track the cost and affordability of specific nutrients and of adequate diets (Håkansson, 2015). Only a few studies have examined the shadow price of individual nutrients using observed market prices to understand the contribution of each nutrient to the total cost of a diet. In Ghana and Tanzania, Masters et al. (2018) found the costliest nutrients to be vitamins B12, A, E in both countries and calcium to be always the most expensive in Tanzania and only sometimes among the costliest in Ghana. In Sweden, Håkansson (2015) found that the cost for adult men and (non-pregnant) women of a diet meeting Nordic nutrition recommendations had not increased more than food prices overall from 1980 to 2012, but that the cost of meeting vitamin D, iron, and selenium recommendations had increased faster (Håkansson, 2015). In applied economics, there is a small literature focused on understanding household demand for nutrients where estimated shadow prices reveal relative preferences for nutrients (Akinleye and Rahji, 2007, Athanasios et al., 1994, Beatty, 2007, Chen et al., 2002, Huang, 1996, Ray et al., 2004, Richards et al., 2012, Shogren, 2006, Shonkwiler et al., 1987, Shrader et al., 2014). Our objective departs from this literature in that we do not aim to explain consumer behavior, but to develop a systematic indicator to track the cost of nutrients in a food system that can be monitored over time to guide policy decisions and evaluate impacts.

Shadow prices from least-cost diets are commonly used outside of human nutrition, such as to guide feed formulation in the livestock industry and in the fields of operations research and environmental management (Balakrishnan and Hung Cheng, 2000, Liu et al., 2009, Saxena and Khanna, 2014). To the best of our knowledge, this is the first study to analyze the shadow price of nutrients in a low-income country’s rural markets to understand the relative efficiency with which a food system provides access to essential nutrients. Modern computing power and software enable this study’s application of least-cost diet research to policy analysis for a nationally representative sample of households over a monthly, five-year time series. Recent computational power thereby expands the possibility to use nutrient shadow price analysis in public policy, human nutrition, and development economics as never before.

## 3 Data & methods

### 3.1 Data

We use household survey panel data matched with monthly retail market food prices, human nutrient requirements, and newly compiled food composition data for Malawi. Table 1 presents the characteristics of the households, food prices, and nutrients in our dataset. Our sample includes 3117 households, monthly price observations in 25 markets for 51 food items, and considers 22 essential nutrients.

**Table 1:** Summary Statistics.

Households*	2013			2016/17			Overall	
	Mean	(SE)		Mean	(SE)		Mean	(SE)
Observations								
Households	1,424			1,693			3,117	
Individuals^†^	7,153			8,221			15,374	
Household size	4.76	(0.12)		4.98	(0.11)		4.90	(0.11)
Age-sex groups cohabitating (max = 22)	4.31	(0.09)		4.51	(0.08)		4.44	(0.08)
Daily food expenditure per cap (2011 US$)	2.35	(0.12)		3.37	(0.93)		3.00	(0.60)
Median	1.72	(0.09)		1.50	(0.06)		1.58	(0.05)
Food as % total expenditure	71.97	(0.80)		70.27	(0.62)		70.89	(0.52)
***Markets & Foods***	2013 – 2017							
				Mean	(SD)			
Foods available per market-month				39.71	(4.82)			
Total foods monitored				51				
Cereals, roots & tubers				9				
Eggs, fish & meat				9				
Dairy				2				
Legumes				5				
Oils & fats				2				
Dark green leafy vegetables				3				
Vitamin A-rich fruits & vegetables				5				
Other fruits & vegetables				9				
Other foods^‡^				7				
Market centers				25				
Nutrients included				22				
Total months				55				

*Population statistics calculated using sampling weights. ^†^ Excludes infants under six months (exclusive breastfeeding).

^‡^Other foods: sugar, biscuits, fried dough, salt, Coca-cola.

#### 3.1.1 Household survey

We use two rounds of the Integrated Household Panel Survey (IHPS) (2013 and 2016/17) to match households with markets and identify individual nutrient needs (age and sex for all household members, occupational data) (National Statistical Office (NSO) [Malawi], 2017). Though households could potentially acquire food from a variety of sources (e.g., own production, gifts, informal transactions), in this study we focus on the opportunity to acquire food from the central market in the household’s district (or traditional authority where there are multiple markets per district) where food prices are regularly monitored in a standardized manner across markets and over time (NSO, 2017, NSO, 2014, NSO, 2012, NSO, 2010). We use two survey rounds because the NSO included the price of more nutrient-dense foods starting in January 2013 (see food list in supplementary materials, Table A-1). We did not have access to the urban centers’ price data and therefore exclude all urban households. Our results are nationally representative for the rural population.

We characterize household composition by aggregated age and sex categories based on the presence of one or more members of each demographic group. Though there are 22 unique demographic groups with different nutrient requirements, to analyze the impact of household composition on the diet cost, we aggregate the categories into eight mutually exclusive groups aligned with the age thresholds for nutrient requirements.

#### 3.1.2 Individual nutrient requirements

We use the individual nutrient requirements defined in the Dietary Reference Intakes (DRIs) (Institute of Medicine of the National Academies, 2011, Institute of Medicine of the National Academies, 2006, National Academies of Sciences Engineering and Medicine, 2019). The selection of requirements and their justification has been presented elsewhere (Schneider et al., 2021b, Schneider and Herforth, 2020). In brief, nutrients included are energy, macronutrients (carbohydrates, protein, lipids), and 19 micronutrients (vitamins and minerals) based on those for which an Estimated Average Requirement (EAR) has been defined and excluding vitamin D, iodine, and molybdenum. We calculate energy needs using the DRIs Estimated Energy Requirement (EER) equations using the median weights and heights by age and sex from the WHO growth standards and references, assuming an ‘active’ level of physical activity for most individuals. Men 14–59 years old engaged in manual labor such as agriculture, day labor, or construction are assumed to have a ‘very active’ level of physical activity. Macronutrient upper and lower bounds are defined by the Acceptable Macronutrient Distribution Range (AMDR) and expressed as a percentage of total calories from each macronutrient. The EAR sets the minimum micronutrient quantity needed and the Tolerable Upper Limit (UL) or Chronic Disease Risk Reduction (CDRR) level sets an upper boundary for 10 of the 19 micronutrients. Additionally, we assume all children 6–23 months are continuing to breastfeed and include only their needs from food sources (Dewey, 2005), and within households where infants are observed we assume their mothers are lactating. We do not make any adjustment for bioavailability because the nutrient constraints imposed result in diets that are by definition adequate in nutrients, and low bioavailability is of concern when diets are inadequate and imbalanced towards plant-based foods (Institute of Medicine of the National Academies, 2006, Institute of Medicine of the National Academies and Institute of Medicine, 2001, WHO, FAO, 2004).

#### 3.1.3 Food prices

The NSO systematically collects monthly food prices for a standardized list of 51 items in 29 rural markets to calculate the Consumer Price Index (CPI) and monitor inflation. Households in the survey live in districts corresponding to 25 of the 29 priced markets. The food list was last defined using the 2010 cross-sectional Third Integrated Household Survey (IHS3) and includes all items accounting 0.02% or more of total household expenditure. Kaiyatsa et al. (2019) describes the data collection, imputation, and cleaning process in detail. While some of the values in the dataset have been imputed by the NSO, these records are not identified. We do not carry out any further imputation of prices and least-cost diet solutions have chosen from among the food items where a price was observed in the given market and month. To understand the relationship between district central market prices and food options and those reported by households, we compared the 45 comparable items between the consumer price dataset and the unit costs reported by households. Though we found prices to differ, we observed no unidirectional patterns (see supplementary materials, Tables A-2 and A-3 for the comparisons). We also note that unit costs reflect both quantity and price, so differences cannot be attributed to different price environments necessarily and the product mix (quality) in the unit costs is unknown while the market prices are for standardized items by design (Gibson, 2019, Gibson, 2016, Gibson, 2013).

#### 3.1.4 Food composition

We match all foods available in the markets to their food composition using the Malawi Food Composition Table (MAFOODS, 2019). The nutrient content of foods varies based on numerous biological factors including natural genetic diversity within and between crop varieties, soil and environmental conditions, processing, storage, and preparation methods (Dickinson et al., 2014, Joy et al., 2017, Joy et al., 2015b, Joy et al., 2015a, Ligowe et al., 2020a, Mlotha et al., 2016). As such, best practices in the nutrition literature emphasize using local tables wherever possible (Charrondière et al., 2013, Greenfield and Southgate, 2012, Micha et al., 2018). We supplement with the USDA food composition data only where a food item match can be confidently made (USDA, 2018).

### 3.2 Household nutrient requirements

Nutrient needs differ by age, sex, reproductive status, and physical activity level, so the lowest cost method for a family to secure a nutritionally adequate diet would be to eat tailored diets that meet each individual member’s own unique needs. In practice, however, families in Malawi commonly eat a shared meal. Infants and young children may be fed separately at other times, but in Malawi as elsewhere most household members eat together and social norms strongly favor food sharing (Gelli et al., 2020, Hjertholm et al., 2019). Defining shared nutrient requirements for a group of people who have different individual needs using nutrient density has its origins in the scientific nutrient requirements literature (Beaton, 1995, Institute of Medicine, 2000). It is also theoretically consistent with Rawls’ maximin principle, to maximize the welfare of the worst-off group in society, or extending to our case to define the household diet that maximizes the welfare of the nutritionally neediest member of the family (Ravallion, 2016, Rawls, 1971, Schneider et al., 2021a).

For a shared meal to meet the needs of each household member, its composition must accommodate variation in their individual requirements (Schneider et al., 2021b, Schneider et al., 2021a). The household’s total energy requirement is the sum of all members’ energy needs. All other requirements depend on which individuals are present and their nutrient density requirements. The person (aged four and above) with the highest nutrient density needs to meet their minimum need defines the household lower bound. Conversely, the member with the lowest nutrient density tolerance to reach their limit defines the household upper bound, which ensures the household diet does not exceed any individuals’ safe intakes. We then define the lower and upper nutrient bounds in terms of quantities by multiplying the nutrient density for each nutrient at each bound by the total household energy need. Finally, we add the individual requirements of each child aged three and below to the household total, under the assumption that each would be fed an individualized diet.

Formally, we define the household nutrient requirements under the presumption of family food sharing by the individual needs of each household’s members (m) for density of each nutrient (j), using the most restrictive of their nutrient density requirements for each upper and lower bound, and meeting total energy needs (E/e):

(1) $${\mathrm{HHLower}}_{j}=\sum_{m}{E_{m}*\max_{m}\{MinimumNeed}_{j,m}/E_{m}\},j=1,\cdots ,19$$

(2) $${\mathrm{HHUpper}}_{j}=\sum_{m}{E_{m}*\min_{m}\{MaximumTolerance}_{j,m}/E_{m}\},j=1,\cdots ,13$$

(3) $${\mathrm{HHE}}_{e}=\sum_{m}E_{m}$$

We term the household bounds as HHE, HHLower, and HHUpper, to distinguish them clearly from the energy balance, minimum needs, and maximum tolerances that have been defined based on biomedical evidence for individuals. The lower bounds used here are defined for 19 nutrients (three macronutrients and 16 micronutrients), by the EAR and AMDR lower bound. Of those, 13 also have upper bounds (three macronutrients and ten micronutrients), defined by their UL, CDRR, and AMDR upper bound. Individual requirements depend on age, sex, physical activity, and maternity status while household needs depend on the number of individuals, their energy requirements, and the specific demographic groups present. Of note, nutrient adequacy is a necessary component of, but alone is insufficient to achieve, a high-quality diet which includes adequate nutrients as well as other characteristics such as balance and variety (FAO, 2020, Trijsburg et al., 2019).

### 3.3 Least-cost diets

Using linear programming, we attempt to solve for a diet minimizing total cost subject to the household nutrient requirement upper and lower bounds and meeting total household-level energy requirements, as specified above. The result provides a cost metric of nutrient adequacy, but it does not return a recommended diet (doing so would require incorporating additional constraints beyond nutrient adequacy such as variety and cultural relevance). This is thus an indicator of food system performance that measures the ability of the food system to facilitate access to a diet that meets the basic bodily needs for health, and which can be compared to the cost for sufficient energy to stay alive (lower cost) and an optimal recommended diet (higher cost) (FAO, 2020, Herforth et al., 2020). The resulting total diet cost per household-month is the Household Cost of Nutrient Adequacy (HHCoNA). Formally, the linear optimization model minimizes total cost over all foods (i) within upper and lower bounds for all nutrients (j) and meets the specified energy budget (HHE). Notation is as for equations (1), (2), and (3), adding data on price for each food (p_i_) and its nutrient contents (a_ij_):

(4) $$\mathrm{HHCoNA}:minimizeC=\sum_{i}p_{i}*q_{i}$$

Subject to:

$$\sum_{i}a_{\mathrm{ij}}*q_{i}\geq {\mathrm{HHLower}}_{j},j=1,\cdots ,19$$

$$\sum_{i}a_{\mathrm{ij}}*q_{i}\leq {\mathrm{HHUpper}}_{j},j=1,\cdots ,13$$

$$\sum_{i}a_{\mathrm{ie}}*q_{i}=HHE$$

$$q_{1}\geq 0,q_{2}\geq 0,\cdots q_{i}\geq 0,for\;all\;foods\;i=1,\cdots 51$$

We solve Eq. (4) for each household every month, using the foods and prices in the market to which they are matched. The primary outcomes for analysis are the percent of household-months where foods with observed prices can meet all nutrient needs, the resulting cost for the household, per person, and per 1000 calories, and the food items in the least-cost diets. Though prices are observed every month, household composition is only observed in each survey round, so we produce a monthly time series for every household with 2013 composition through December 2015 and 2016/17 composition from January 2016 forward.

### 3.4 Nutrient shadow prices

A useful feature of least-cost diets is that the calculation also identifies the shadow price for each nutrient, which measures the sensitivity of cost to small changes in that requirement. Formally, each shadow price is the Lagrangian multiplier in the corresponding (dual) solution to the optimization problem. They are calculated by rearranging Equation (4) as:

(5) $$\mathrm{HHCoNA}:minimizeC=\sum_{i}p_{i}*q_{i}$$

Subject to:

$$-\left(\sum_{i}a_{\mathrm{ij}}*q_{i}\right)+{\mathrm{HHLower}}_{j}\leq 0,j=1,\cdots ,19$$

$$\sum_{i}a_{\mathrm{ik}}*q_{i}-{\mathrm{HHUpper}}_{j}\leq 0,j=1,\cdots ,13$$

$$\sum_{i}a_{\mathrm{ie}}*q_{i}-HHE=0$$

$$q_{1}\geq 0,q_{2}\geq 0,\cdots q_{i}\geq 0,for\;all\;foods\;i=1,\cdots 51$$

Then defining a non-negative dual variable for the inequality constraints and an unrestricted variable for the equality constraint gives:

(6) $$\lambda_{1}(-\left(\sum_{i}a_{\mathrm{ij}}*q_{i})+{\mathrm{HHLower}}_{j}\right)$$

(7) $$\lambda_{2}\left(\sum_{i}a_{\mathrm{ik}}*q_{i}-{\mathrm{HHUpper}}_{j}\right)$$

(8) $$\lambda_{3}\left(\sum_{i}a_{\mathrm{ie}}*q_{i}-HHE\right)$$

Where each λ is the Lagrangian multiplier indicating the incremental amount that the objective function changes for a one-unit increase in the nutrient requirement (HHLower, HHE) or upper bound limit (HHUpper). A non-zero Lagrange multiplier means that the constraint is binding; a change in the right-hand side value would change the result. A zero-value multiplier means the opposite, that the constraint is met automatically when meeting others and would not change the total cost result if it were to move up or down on the margin (Håkansson, 2015, Lahaie, 2015). In almost all cases, the number of non-zero shadow prices will be equal to the number of unique foods included in the least-cost diet.

The shadow price is interpreted as the amount that the total diet cost increases (decreases at the upper bound) for a one-unit increase in the constraint. We transform each marginal cost into semi-elasticities because measurement units vary, interpreted as the amount the total diet cost changes for each percentage increase in the amount of that nutrient required (lower bound) or allowed (upper bound). Lower bounds will have a positive sign for all nutrients whose minimum constraints are binding on the margin. Upper bounds reflect the cost decrease if upper bound constraints were relaxed, expected to have a negative sign for all upper limits that are binding on the margin.

### 3.5 Policy scenarios

We model eight policy scenarios that illustrate how government and private sector actions could improve access to nutritious diets, choosing a range of realistic actions. In earlier work we identified the nutrients in shortest supply to be phosphorus, riboflavin, lipids, selenium, and vitamin B12 (Schneider et al., 2021b).^1^ In a preview of our results below, we found that riboflavin, vitamin B12, and selenium and to a lesser extent vitamin E and niacin are also the nutrient requirements to which diet costs are most sensitive. As such, these nutrients guided our selection of policy scenarios.

We identified the best food sources of the multiple nutrients lacking in diets and expensive in Malawi’s markets to be fresh and powdered milk (riboflavin, lipids), eggs (riboflavin, B12, lipids), fish (B12, niacin), cooking oil (vitamin E, lipids), and groundnuts (vitamin E, lipids, niacin) (MAFOODS, 2019). Given the current availability and prices, we selected the following scenarios to reflect possible changes in availability, price, or both, for products in value chains that could be prioritized for investment. If the constraints to finding an affordable diet are that nutrient-dense foods are less available and cost more, these scenarios will provide *ex ante* evidence for which value chains would be most promising investments to lower the cost of a nutrient adequate diet. In addition to these general and synthetic scenarios, we also selected a specific intervention: selenium soil biofortification of maize. This intervention has been tested and shown to be a pragmatic and cost-effective way to increase selenium availability in Malawi and is of interest to policymakers (Chilimba, 2011, Chilimba et al., 2014, Chilimba et al., 2012a, Chilimba et al., 2012b, Joy et al., 2019, Ligowe et al., 2020b). Our initial motivation to include this very qualitatively different scenario was its policy relevance, however doing so ultimately revealed the challenge limiting the ability to identify an adequate diet in so many household-months. Further background and information on the policy scenarios is provided in the supplementary materials (section B). In brief, the scenarios are:

1. **Lower price of eggs**: Eggs are available in all markets and months and at A) 10%, B) 15%, and C) 20% lower price than observed in that market and month under baseline conditions.
2. **Increased availability of dried fish**: Dried fish are available in all markets and months at the median price currently observed.
3. **Increased availability and lower price of groundnuts**: Groundnuts are available in all markets and months and at a 10% lower price than observed in that market and month under baseline conditions.
4. **Lower price of fresh milk**: Fresh milk is available in all markets and months and at a 10% reduction than observed in that market and month under baseline conditions.
5. **Increased availability of powdered milk**: Powdered milk is available in all markets and months at the median price currently observed.
6. **Soil biofortification (for maize)**: Soil biofortification with selenium is applied at the production stage for all maize in the market, and resulting in maize grain and flour with selenium content as observed in field trials (Chilimba, 2011, Chilimba et al., 2014, Chilimba et al., 2012a, Chilimba et al., 2012b, Joy, 2020a, Joy et al., 2019, Ligowe et al., 2020b).

Eggs and fresh milk are already available in most markets and months, while fish, groundnuts and powdered milk are unavailable in a large proportion of markets and months (see supplementary materials, Figures B-2 to B-6). Where the items are not currently available and the scenario assumes universal availability and a price reduction (eggs, fresh milk, groundnuts), we first impute the missing price as the median price in that same month over remaining markets where it is present. We then reduce the price by the specified percentage relative to the observed or imputed price in each market and month. For the selenium biofortification scenario, we adjust the food composition of maize grain and flours based on the amount that resulted from the experimental intervention; 11.3 mcg selenium per 100 g edible portion in whole maize grain and whole grain flour, and 5.1 mcg in degermed and dehulled flour, a 55% reduction in selenium content due to the removal of the germ and bran (Chilimba, 2011, Chilimba et al., 2014, Chilimba et al., 2012a, Chilimba et al., 2012b, Joy, 2020a, Joy et al., 2019, Ligowe et al., 2020b). This amount was guided by conventional fortification decision process that considers the quantity of the food item consumed on average, required amounts, and upper limits not to be exceeded and identifies a fortification level that will be both adequate and safe when consumed in typical amounts by the whole population (WHO and FAO, 2006). These trials are being conducted under real-world conditions on-farm (Joy, 2020b, Joy, 2020a, Joy et al., 2019).

The first seven scenarios are stylized outcomes that could result from numerous policy actions. For example, increased production of fresh milk and eggs and value chain efficiency could arise from investments to improve animal health and productivity or might come in response to increased demand where supply increases are possible with current technology and herd/flock size. For fish, investments in aquaculture could increase production while storage infrastructure could potentially smooth stocks throughout the year. The availability and price of groundnuts could be affected by breeding and agronomic investments, as well as investments in storage. In addition to the policy and business feasibility of these scenarios, they are also likely to be acceptable to consumers. Current evidence suggests that consumers like milk, eggs, fish, and groundnuts and would prefer to eat more at lower prices but consider these foods expensive (Akaichi et al., 2016, Ecker and Qaim, 2011, Gelli et al., 2020, Simtowe et al., 2016, Stewart et al., 2019). This evidence supports that these foods fit into cultural dietary traditions, but increasing access in terms of availability and affordability is necessary but likely on its own insufficient to result in nutritionally meaningful increases in consumption (Fanzo, 2014, Ridoutt et al., 2019).

## 4 Results

### 4.1 Shared household nutrient requirements

**Fig. 1:**
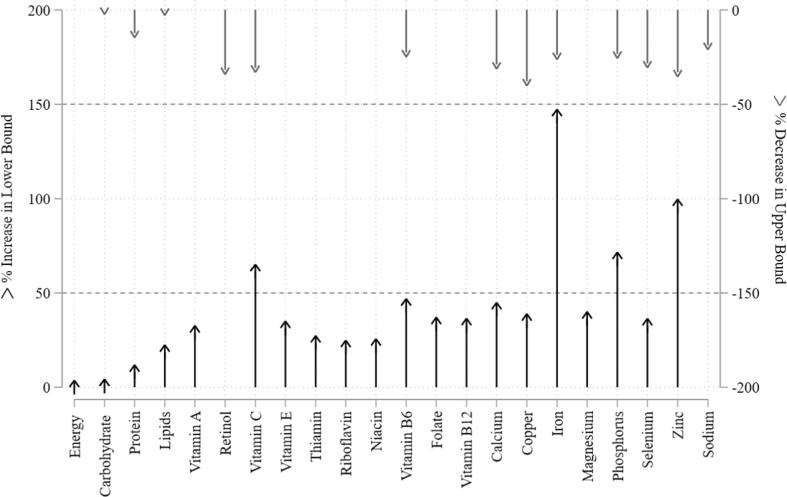
Percent Difference in Nutrient Bounds from Individual Diets to Household Sharing, Population statistics calculated using sampling weights.

Fig. 1 illustrates how the shared diet changes the total nutrient requirements relative to what would be sufficient to meet each person’s own needs as scientifically defined (DRIs) based on age, sex, physical activity, and maternity status. For example, in a family of five people (breastfeeding mother, father, son 13-months, daughters seven and nine years old), the nine-year-old daughter has the highest nutrient density need for iron, so the quantity she needs to meet her minimum needs is the same amount she would consume with the shared diet while all other members will eat the amount of iron per calorie that she needs, in the total amount that provides enough calories to meet their own energy need, others would eat more than minimum needed but not exceed their upper limit. In Fig. 1, the average amount needed per nutrient to meet individual needs over the whole population, weighted by population shares, is compared to the amount individuals would consume eating their respective household’s shared diet in proportion to individual energy needs. The arrows represent the magnitude of the difference with those facing upward reflecting minimum requirements and downward are upper limits.

The shared requirement results in a higher need (50% or more) for vitamin C, iron, phosphorus, and zinc. On the upper bound, copper, zinc, retinol, and vitamin C have lower limits by 30% or more under sharing. Taken together, the range tightens the most for iron and zinc. Table D-1 (supplementary materials) presents the full results.

### 4.2 Base case

Table 2 presents the least-cost diet and shadow prices resulting from currently observed food item availability and prices. We find the adequate shared diet costs $2.32 per person per day ($2.23 at median) over the period January 2013–July 2017, well above the international poverty line of $1.90. We have shown in related research that this cost makes the diet unaffordable within current food budgets for 80% of the population (Schneider et al., 2021a). Further, there is no solution to the linear programming problem 40% of the time. The high cost and large proportion of infeasible results motivate our further exploration below.

**Table 2:** Diet Cost, Feasibility and Nutrient Semi-Elasticities.

	Mean	(SE)
Household cost per day (2011 US$)	10.06	(0.28)
Per 1,000 kcal	1.21	(0.01)
Per person	2.32	(0.03)
Diet Feasible (% HH-Months)	59.37	(1.58)
Semi-elasticities – Lower Bound*		
Riboflavin	2.57	(0.19)
Niacin	0.01	(0.00)
Vitamin B12	0.14	(0.01)
Selenium	0.01	(0.00)
Semi-Elasticities – Upper Bound*		
Copper	−0.24	(0.01)
Iron	−0.01	(0.00)
Zinc	−0.01	(0.00)

Population statistics calculated using sampling weights.

Heteroskedasticity robust standard errors clustered at the enumeration area level. Outliers, defined as households with a HHCoNA more extreme than 1.5 times the IQR, excluded. *Only non-zero shadow prices are shown.

We find riboflavin is the most expensive nutrient. A 1% increase in riboflavin requirement increases the diet cost by an average $2.57 per day– greater than the cost of adding an entire additional family member.^2^ The next most costly nutrient pales in comparison to riboflavin; the least-cost diet rises $0.14 for 1% B12 requirement increase.^3^ The diet cost rises $0.01 for a 1% increase in niacin and selenium requirements.^4^

Regarding the upper bounds, we find that copper and (to a lesser extent) iron are binding constraints, meaning that a lower total cost could be achieved if the upper limits on these minerals were relaxed. In other words, taking copper as an example, in the process of solving the linear optimization, the copper upper bound is reached before other nutrient requirements have been satisfied, which then reduces the food item options to provide those remaining nutrients to only foods that have no copper. Allowing 1% more copper into the diet would decrease the total cost by $0.24 per household per day.^5^

#### 4.2.1 Influence of household size & composition

Since the household adequate diet is defined by the combination of different types of members, it is possible that only certain compositions account for the high cost or infeasible results. If this is the case, the policy remedies to improve access to affordable diets might better be targeted. However, if the most common configurations of household members face the highest diet costs, policy remedies that operate throughout a food system would be efficient. Table 3 illustrates the diet feasibility and cost by household composition for the five most frequent compositions, and the most and least feasible and costly. We see household composition drives feasibility more than the cost, and that when the diet is feasible the variation in cost per 1000 calories for the most common compositions differs by at most $0.13.

**Table 3:** Household Composition, Diet Feasibility, and Diet Cost.

		Households (%)		Feasibility (%)			Cost per 1,000 kcal (2011 US$)	
				Mean	SE		Mean	SE
Most common compositions*								
Older child(ren), adolescent(s), male and female adults		15.6		38.67	(2.78)		1.32	(0.02)
Older child(ren), male and female adults		12.0		69.93	(2.61)		1.28	(0.02)
Young child(ren), older kid(s), male and female adults		8.7		82.02	(2.36)		1.19	(0.02)
Young child(ren), older child(ren), male and breastfeeding female adults		7.0		55.04	(2.65)		1.31	(0.02)
Young child(ren), older child(ren), adolescent(s), male and female adults		5.8		61.58	(2.10)		1.28	(0.02)
Total		49.4		58.99	(1.85)		1.27	(0.01)
***Feasibility Extremes***								
**Most:** Older child(ren), adult male(s)		0.2		100.00	(.)		0.89	(0.03)
**Least:** Adolescent(s), male and female adults, older adult(s)		0.4		3.48	(3.27)		1.33	(0.02)
***Cost Extremes***								
**Most:** Young child(ren), adult female(s)		0.4		44.16	(11.35)		1.96	(0.05)
**Least:** Adolescent(s)		0.1		86.27	(11.99)		0.74	(0.17)

Population statistics calculated using sampling weights. Composition types sorted by frequency observed.

* Most common compositions comprise 50% of the population.

Definition of age groups aggregates the age groups in the DRIs as follows: Young children = 3 and below, Older children = 4–13, Adolescent = 14–18, Adult = 19–69, Older adult = 70 and above.

Full results for all compositions observed in five or more households provided in the supplementary materials, Table C-1.

**Fig. 2:**
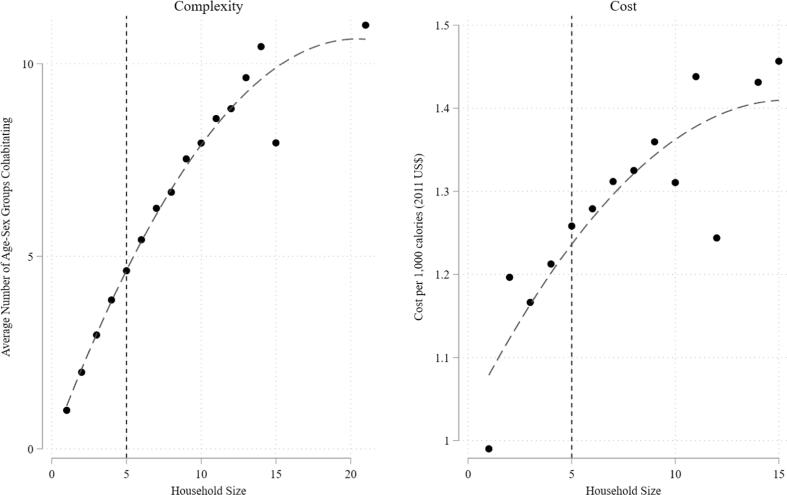
Household Size, Composition, and Cost per 1,000 calories, Population statistics calculated for survey weights. Vertical line reflects mean household size of five people. Fit line reflects quadratic fit.

Fig. 2 illustrates the relationship between household size, composition, and resulting diet cost. Each additional household member of a different type (age, sex, or maternity status) than already present could increase the required diet quality. We observe household composition complexity (number of different types) increases with household size up to about ten members, after which additional members are less likely to change the nutrient density requirements. In other words, larger households are not just scaled up smaller households, but they are likely to require greater nutrient density because they also have more complex membership.

Fig. 2 also illustrates that as households grow in size and complexity, higher income is necessary to purchase 1000 calories of the diet that meets the household’s basic nutrient needs. Economists have long been concerned with economies of scale in household consumption, which are critical to comparing levels of welfare across households of differing size and composition to draw conclusions about poverty and inequality (Browning et al., 2013, Deaton and Paxson, 1998, Gibson, 2002). Our results extend this literature taking a cost of basic needs approach, which suggests there exists a diseconomy of size, resulting from the diversity in nutrient requirements when household complexity increases, at least where families share food.^6^

We next consider whether the shared nutrient requirements drive the nutrient shadow prices, meaning that using individual requirements instead would find different nutrients to be the costliest or binding. Bearing in mind that the costliest nutrients (riboflavin, B12, niacin, selenium) on the lower bound are not among those where the family food sharing increased the requirement or tightened the range by a larger percent than for most other nutrients (nutrients that did were iron, zinc, vitamin C, phosphorus), we argue our results are likely an accurate reflection of the relative cost of nutrients in a food system on the lower margin.

Of the binding nutrients on the upper bound (copper, iron, zinc), iron and zinc are among those most altered by defining a shared household nutrient requirement. Our estimate of how much avoiding excess intake drives up the diet cost may be biased upwards for these nutrients. In other words, some individuals could tolerate more copper, iron, or zinc in their diet, and find a lower-cost diet without exceeding their individual upper limit but are constrained by the lower nutrient density tolerated by other family members. However, where family food sharing is the dominant cultural norm the benchmark diet costed for policy information arguable should be one that would not risk exposing any member to a potential toxicity when eating an adequate share of their family meal.

### 4.3 Policy scenarios

**Fig. 3:**
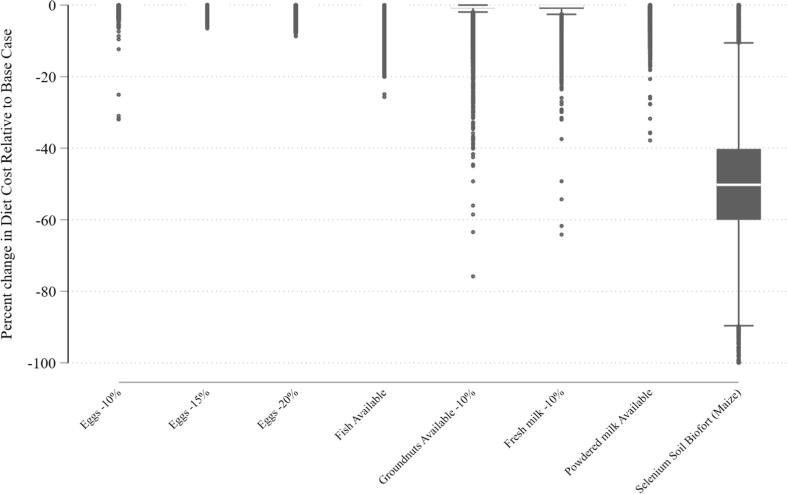
Change in Diet Cost Relative to Base Case, by Household and Month, Population statistics calculated using sampling weights. Outliers (defined above) excluded.

Policy simulations reveal the striking finding of this paper that selenium is the nutrient constraining the availability of nutrient-adequate diets. Fig. 3 demonstrates the impact of each simulated scenario on the diet cost for the same households, markets, and months where it was feasible under the base case (current conditions). For the eggs, fish, and powdered milk scenarios, the difference in diet cost is negligible. This suggests that even with the price and availability change, those foods remain more costly sources of nutrients than the alternatives already selected into the diets under the base case, or that some other nutrient limitation still leaves costlier foods entering the diet. The fresh milk and groundnut scenarios result in at most a 1% decrease. Selenium soil biofortification stands out clearly with a median decrease in diet cost of 50% and half of household months demonstrating a diet cost decrease between 40% and 60%. This suggests that selenium is the nutrient constraint that is driving up the cost of the diet, though not necessarily because selenium itself is not available or costly.

Table 4 presents the full results, illustrating that most of the scenarios have no meaningful impact on any of the metrics. Decreasing the cost of fresh milk by 10% substantially reduces the shadow cost of riboflavin but does not change the other metrics. Soil biofortification of maize with selenium, not only reduces the total diet cost almost in half but also results in near universal feasibility of the diet (95% of household-months become feasible), again underscoring that meeting selenium requirements without selenium available in maize was limiting the feasibility of the diet.

**Table 4:** Cost, Feasibility and Nutrient Elasticities by Policy Scenario.

	Base case			Eggs −10%			Eggs −15%			Eggs −20%			Fish	
	Mean	SE		Mean	SE		Mean	SE		Mean	SE		Mean	SE
Household cost/day (2011 US$)	10.07	(0.28)		10.06	(0.28)		10.06	(0.28)		10.06	(0.28)		10.05	(0.28)
Per person	2.32	(0.03)		2.32	(0.03)		2.32	(0.03)		2.32	(0.03)		2.32	(0.03)
Diet Feasible (% HH-Months)	59.40	(1.58)		59.43	(1.58)		59.40	(1.58)		59.41	(1.58)		59.41	(1.58)
Semi-Elasticities – Lower Bound*														
Riboflavin	2.57	(0.19)		2.79	(0.24)		2.78	(0.24)		2.76	(0.24)		2.58	(0.19)
Niacin	0.01	(0.00)		0.01	(0.00)		0.01	(0.00)		0.01	(0.00)		0.01	(0.00)
Vitamin B12	0.14	(0.01)		0.14	(0.01)		0.14	(0.01)		0.13	(0.01)		0.13	(0.01)
Selenium	0.01	(0.00)		0.01	(0.00)		0.01	(0.00)		0.01	(0.00)		0.01	(0.00)
Semi-Elasticities – Upper Bound*														
Copper	−0.24	(0.01)		−0.24	(0.02)		−0.24	(0.02)		−0.24	(0.02)		−0.24	(0.01)
Iron	−0.01	(0.00)		−0.01	(0.00)		−0.01	(0.00)		−0.01	(0.00)		−0.01	(0.00)
Zinc	−0.01	(0.00)		−0.01	(0.00)		−0.01	(0.00)		−0.01	(0.00)		−0.01	(0.00)
	Base case			Groundnuts			Fresh milk			Powdered milk			Soil fortification	
Household cost/day (2011 US$)	10.07	(0.28)		10.03	(0.28)		10.13	(0.27)		10.06	(0.28)		5.91	(0.18)
Per person	2.32	(0.03)		2.30	(0.03)		2.31	(0.03)		2.32	(0.03)		1.22	(0.02)
Diet Feasible (% HH-Months)	59.40	(1.58)		59.92	(1.54)		60.88	(1.58)		59.46	(1.58)		94.94	(0.52)
Semi-Elasticities – Lower Bound*														
Riboflavin	2.57	(0.19)		2.59	(0.20)		1.99	(0.16)		2.37	(0.18)		2.62	(0.17)
Niacin	0.01	(0.00)		0.01	(0.00)		0.01	(0.00)		0.01	(0.00)		0.00	(0.00)
Vitamin B12	0.14	(0.01)		0.15	(0.01)		0.13	(0.01)		0.14	(0.01)		0.10	(0.01)
Selenium	0.01	(0.00)		0.01	(0.00)		0.01	(0.00)		0.01	(0.00)		0.00	(0.00)
Semi-Elasticities – Upper Bound*														
Copper	−0.24	(0.01)		−0.24	(0.01)		−0.23	(0.01)		−0.24	(0.01)		−0.01	(0.00)
Iron	−0.01	(0.00)		−0.01	(0.00)		−0.01	(0.00)		−0.01	(0.00)		−0.00	(0.00)
Zinc	−0.01	(0.00)		−0.01	(0.00)		−0.01	(0.00)		−0.01	(0.00)		−0.01	(0.00)

Population statistics calculated using sampling weights. Heteroskedasticity robust standard errors clustered at the enumeration area level. Outliers (defined above) excluded.

* Only non-zero shadow prices are shown.

Biofortification nearly eliminated the shadow cost of selenium, as might be expected, but it also reduced that of B12 by one third and niacin approximately 80%. This is explained by legumes supplying a large share of selenium under the base case scenario. Though legumes contain carbohydrates, protein, niacin, energy, and other nutrients in addition to selenium, they are likely to be a more costly source of these other nutrients than alternatives. However other food sources of selenium (leafy greens, vitamin A-rich fruits) do not contain as much energy, carbohydrates, or any protein so the total diet cost would be higher (and potentially not feasible without exceeding other upper bounds) if energy and macronutrients came from other sources and selenium from these fruits and vegetables.

Most interestingly, selenium biofortification nearly eliminates the shadow price of copper and reduces the other upper bound constraints. This means that the greater availability of selenium in maize prevents the need to meet selenium and other requirements from foods that are dense in copper, or from more expensive foods that are not dense in copper and enter the diet to supply remaining nutrients while staying within the copper upper bounds. While the selenium shadow cost was non-zero in the base case, it was small in practical terms at only $0.01, underscoring that the costliest nutrients in the market when an adequate diet is feasible may not be the ones limiting feasibility to begin with.

**Fig. 4:**
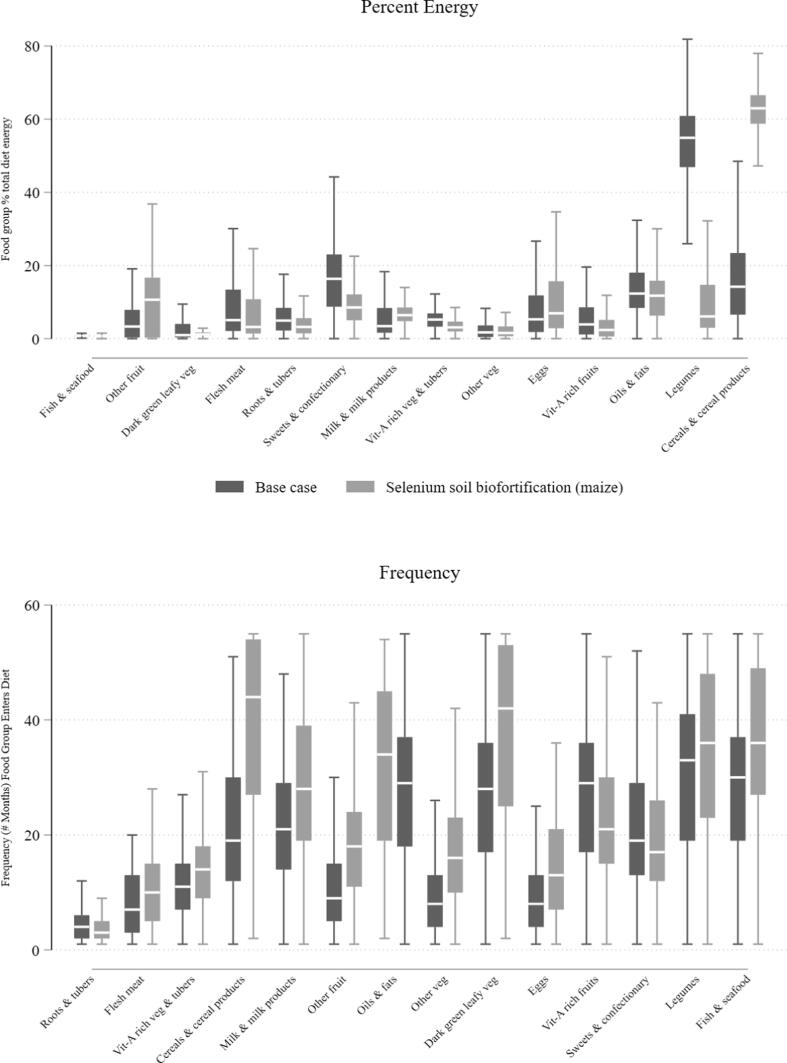
Difference in Diet Composition by Food Group, Population statistics calculated using sampling weights. Outliers (defined above) excluded. Sorted from left to right on y-axis variable.

To understand why the diet cost changes so dramatically under soil selenium biofortification, we examine the composition of the diet by food group under the base case and selenium biofortification scenarios. The top panel of Fig. 4 illustrates the difference in percent of energy from each food group. It shows the greatest difference to be the share of legumes and cereals in the diet. In the base case, a large percent of energy (>50% at the median) comes from legumes and a lower share from cereals (10%) than would be expected given the latter are generally the cheapest source of energy and carbohydrates. The stylized intuition behind this result is that the base case diet gets energy and carbohydrates from a more expensive source of those nutrients (legumes vs. cereals) because the legumes must enter to supply other nutrients. Even though they may be a more expensive source of energy and carbohydrates, they would enter instead of cereals because they are the least expensive source of the additional nutrients present in legumes but not sufficiently present in cereals: protein, lipids, vitamin E, riboflavin, folate, copper, selenium, and zinc. When the selenium shortage is alleviated through soil biofortification of maize, the diet gets much more energy from cereals, making it cost less. Eggs, meat, oils, and fats supply similar amounts of dietary energy across scenarios.

The lower panel of Fig. 4 shows the distribution of total frequency that each food group appears in households’ diets. Almost all food groups appear more frequently under the biofortification scenario because there is a solution for nearly every household in every month. The key insight is the clear divide with certain food groups rarely offering the lowest cost source of essential nutrients under either scenario: roots and tubers, other fruits and vegetables, meat, eggs, and vitamin-A rich fruits.^7^ Thus the selenium scenario serves several functions in this deductive analysis. First, it illustrates how selenium, and its co-occurrence with copper, constrains the feasibility of a nutrient-adequate diet in Malawi given current soil composition. But second, it shows which nutrient-dense food groups are more often in the least-cost diet. Investments to increase the availability and lower the cost of these foods in addition to alleviating the selenium constraint through biofortification, would likely be the most promising investments in the short-term to bring the nutrient-adequate diet into affordable reach. Such food groups include dark green leafy vegetables, legumes, and fish.

Modeling scenarios aids in uncovering the sensitivity of the level estimates to changes in a single parameter. As a data envelopment technique, linear programming results are only sensitive to changes in the data that affect the optimization on the margin, i.e., the price or nutrient content of the lowest cost item. We find that limited changes to prices and availability for eggs, powdered milk, and fish had no impact on the results, indicating those foods supply nutrients available at lower cost in other foods, and the change in price or availability was not sufficient to alter that relative costliness. Where the change in price affected the item already selected as the lowest cost source of a nutrient, one could expect a more linear relationship with the change in diet cost. However, keeping in mind that all foods contain multiple nutrients, the change in price could alter the relative nutrient costs across foods resulting in a shift in the composition of the food basket and a non-linear relationship between a change in the item price and the diet cost. We emphasize overall that the value of these methods lies not in the level estimate they produce but in the comparison over time, space, and under different policy conditions, when collected and estimated systematically.

## 5 Conclusions

In this paper, we demonstrate how food prices, and the availability of food items can be used to diagnose how well a food system currently facilitates access to nutrient-adequate diets for all members of households who typically eat together. We calculate the cost of an adequate diet – one that is sufficient in total energy, meets minimum micronutrient limits, is balanced in macronutrients, and does not exceed upper bounds – for rural Malawian households in their nearest district central market, accounting for the higher nutrient density required when families share food.

We find that at food availability and prevailing prices from January 2013–July 2017, the diet is available in 60% of all household-months at an average cost of $2.32/person/day. Riboflavin is the costliest nutrient; the cost of an adequate diet increases $2.57 on average for a 1% increase in riboflavin requirement. Vitamin B12 is the next most costly, increasing the diet cost by $0.14, on average, for a 1% increase in the requirement. Niacin and selenium also raise the diet cost, but only by $0.01, on average, for a 1% increase in their requirement. At the same time, allowing 1% more copper into the diet would reduce the cost by $0.24 on average, with a $0.01 reduction for similar relaxation of the upper limits on iron and zinc. We find household composition drives infeasibility, but that cost variation across compositions where there is a feasible diet is small in practical terms. We demonstrate a positive linear relationship between household size and the diet cost per 1000 calories, irrespective of composition, revealing an apparent diseconomy of size in the cost of meeting basic needs as household size grows because the complexity of household nutrient needs also increases.

Our findings highlight that the diet cost and nutrient shadow price analysis can only reveal how well a food system facilitates access to a nutrient-adequate diet when the available food items can be combined at some cost to meet the nutrient requirement constraints. However, when the diet is not feasible, scenario modeling is an effective way to identify the barriers. Though riboflavin, B12, and copper emerged with the highest shadow prices and therefore appeared to be driving the cost, we clearly identified that selenium is the constraining nutrient on feasibility. Introducing selenium soil biofortification of maize, we found would increase the percent of household-months where the diet is feasible from 60% to 95% and cut the average cost nearly in half. Selenium biofortification would eliminate the specific constraint imposed by the co-occurrence of selenium and copper in many foods where incompatible amounts would be needed to stay within the nutrient requirement bounds for both nutrients.

We note that the food composition data have some gaps, which may be consequential for our conclusions regarding selenium. There are more food items with missing selenium data than for any other nutrient. We assume the food contains no selenium, but if wrong, then more selenium may be available than we estimate. Our estimate of the diet cost might then be higher than the truth and estimated feasibility lower than the truth, but only if items missing selenium data that actually contain selenium would have been selected into the least-cost diet had the information about the selenium quantity been present. However, we justify our assumption that zero selenium is a reasonable assumption where data are lacking by the findings of Joy et al., 2015a, Joy et al., 2015b, Phiri et al., 2019 who measured the presence of selenium in soils, foods, and deficiency in the population and concluded there is little selenium present and available to consumers and high levels of deficiency. However, additional food composition analysis is warranted before supporting a soil biofortification program.

Our study contributes to a large body of research on least-cost diets. We make a unique contribution by estimating requirements for whole households who commonly eat together from shared meals, ensuring that the specific needs of every individual in the household are met. We argue this is not only realistic, but also serves the policy information need of decision-makers beyond the health sector who are generally concerned with households. Furthermore, using observed household composition results in an estimate of the diet cost that reflects the relative nutrition requirements of all age and sex groups in their representative proportions relative to the population pyramid. Though a similar population level metric could be computed as a weighted average of individual sub-group diet costs, doing so would not reflect the typical composition of households and the fact that families share meals.

Ours is among few studies of nutrient shadow prices particularly in low-income countries, which we use to identify the nutrients driving the total diet cost. This is arguably an underutilized tool in the human nutrition and food policy domain, though it only reveals information about a food system when the diet meeting specified constraints is feasible. We further extend the existing literature with the only analysis to date, to the best of our knowledge, using scenario simulations to identify the drivers of both diet cost and feasibility and to model realistic policy scenarios to assess the potential effects on total diet cost. Future research could investigate alternative methods such as using average unit costs from the household survey as a proxy for prices in order to capture the full range of foods consumed by households (including those from own production) and to increase the spatial granularity to understand more localized realities. Investigating tradeoffs and the costs and benefits of different policy options also present important questions for future research.

We hope our study demonstrates a salient and useful metric to diagnose the needs of the population and the ability of a food system to meet those needs. One clear policy implication for Malawi emerges: rural households are not reliably able at present to access a nutritionally adequate diet. We demonstrate that the higher level of diet quality required when families share food may not be possible given current food items available. Even when available, the cost per capita exceeds the international poverty line under which many rural Malawians live (Schneider et al., 2021a, World Bank, 2021). However, we also demonstrate that selenium biofortification of maize, the country’s main food security crop and the main focus of much of its food security policy and budget, could dramatically reduce the cost of nutritious diets. This is feasible, in part, through the existing Affordable Inputs Program (formerly known as the Farm Input Subsidy Program) which is an existing channel through which selenium-fortified fertilizer could be made available to farmers (Chilimba et al., 2012b, Joy, 2020a, Joy, 2020b). Beyond Malawi, the approaches we demonstrate in this paper can augment the growing toolkit of least-cost diet methods for food and nutrition policymaking and contribute to the evidence-based selection of policy options that can support food systems’ transformations to become nutrition-sensitive and increase access to affordable, nutritious diets for all.

## CRediT authorship contribution statement

**Kate R. Schneider:** Conceptualization, Methodology, Software, Formal analysis, Data curation, Writing – original draft, Writing – review & editing.

## Declaration of Competing Interest

The authors declare that they have no known competing financial interests or personal relationships that could have appeared to influence the work reported in this paper.

## Footnotes

1 ^1^ Figure B-1 in the supplementary materials presents the energy-adjusted adequacy ratios of current household diets relative to their shared needs. Those data reflect the extent to which observed diets are dense enough in each nutrient to meet the needs of every family member, regardless of whether the diet contained sufficient energy or not.

2 ^2^ Riboflavin is found in animal-source and plant-based foods, and there are many items rich in riboflavin in Malawi’s retail markets (supplementary materials, Table A-4). Riboflavin may not commonly be considered a nutrient of public health concern, but serious health consequences of deficiency are possible; it is needed for energy metabolism, growth and development, and the metabolism of other compounds in the body including those that activate vitamin A and metabolize other B vitamins. Deficiency causes inflammation and cracking of the skin, affects the red blood cells (causing anemia, fatigue, confusion, headaches), and is especially dangerous for pregnant women (Institute of Medicine of the National Academies, 2006).

3 ^3^ B12 is only found in animal source foods and deficiency causes a form of anemia because it is needed to metabolize proteins, as well as certain fatty acids. Importantly, it is also necessary to metabolize folate, so a B12 deficiency can cause a folate deficiency even if sufficient folate is consumed in the diet. Folate insufficiency in a mother at conception causes neural tube defects.

4 ^4^ Niacin is found in grains and in animal source foods. It is required for the basic cycle of energy metabolism and deficiency causes problems of the skin, dementia, diarrhea and can lead to death. Selenium is found in animal source and plant-based foods but the selenium content in plants is driven entirely by the levels of selenium in the soils where the foods were grown. It is an important antioxidant and is required to synthesize thyroid hormones. Deficiency does not cause a specific disease, but it is associated with the development of heart problems and some evidence in animals suggests that selenium adequacy is protective against the harmful effects of exposure to aflatoxin, a carcinogenic mycotoxin known to be widespread in maize and groundnuts in Malawi (Diaz Rios, 2013; IARC Working Group on the Evaluation of Carcinogenic Risks to Humans, 2009; Institute of Medicine of the National Academies, 2006; Limaye et al., 2018; Matumba et al., 2014; Misihairabgwi et al., 2017; Monyo et al., 2012; Shi et al., 1994, 2012; Wang et al., 2013; Zhao et al., 2019).

5 ^5^ Though copper toxicity is considered rare, it causes liver damage and can cause hemolytic anemia (bursting of red blood cells) so understanding whether this constraint could be relaxed in a meal sharing context requires biomedical nutrition study (Institute of Medicine of the National Academies, 2006).

6 ^6^ Theory of household public goods posits that a larger household will have higher per capita expenditure on private goods than a smaller household of equivalent per capita resources (Barten, 1964; Deaton and Paxson, 1998). Empirical evidence, however, appears to present a paradox whereby holding household composition constant, larger households have lower per capita food expenditure than smaller households of equivalent income (Deaton and Paxson, 1998; Logan, 2011). Differing prices (bulk discounts and more expensive, smaller, more frequent quantities as a rationing device) and measurement error have been put forth and supported empirically (Dillon et al., 2021; Gibson, 2002; Gibson et al., 2017; Gibson and Kim, 2018). Our analysis has shown that the cost of meeting basic nutrient needs differs with both household size and composition, due to needs for higher diet quality by certain age and sex groups. Therefore where the objective is to estimate or identify households living in poverty, an alternative approach would be to compare observed incomes to flexible poverty lines that incorporate variation in nutrient requirements by household size and composition. Allen (2017) demonstrates how least-cost diets can be used to define the food component for internationally comparable poverty lines, and our approach could extend that application with a justified selection of nutrients necessary for optimal growth and long-term health and the nutrient density approach to define household diet quality to estimate poverty lines flexible to household composition.

7 ^7^ To further contextualize these results, supplementary materials Table A-1 contains all the food items in the price dataset by food group, Table A-4 all the items containing each nutrient (quantity per 100g edible portion and by nutrient density), and Table A-5 the median price per kg (2011 $US).
