# Supplementary material for: Nationally representative estimates of the cost of adequate diets, nutrient level drivers, and policy options for households in rural Malawi
Source: Food Policy. 2022 Nov;113:102275. doi: 10.1016/j.foodpol.2022.102275 (PMC9763653; doi:10.1016/j.foodpol.2022.102275)
Supplement: Supplementary Data 1 [file mmc1.docx]

**Supplementary Material**

1. **Food Items Identification and Nutrient Composition**

Table A-1. Food Items by Food Group in Price Dataset

| **Food Group** | **Items** |  | **Food Group** | **Items** |
| --- | --- | --- | --- | --- |
| Cereals &  Cereal  Products | Maize flour (dehulled) |  | Vitamin-A rich fruits | Mangoes |
|  | Maize flour (whole grain) |  |  | Oranges |
|  | Maize grain |  |  | Papaya |
|  | Maize grain, Admarc |  |  | Tomatoes |
|  | Rice grain |  | Vit-A rich Vegetables | Pumpkin |
|  | White bread |  | Other Fruits | Avocado |
| Dark Green Leafy  Vegetables | Chinese cabbage |  |  | Banana |
|  | Pumpkin leaves |  |  | Guava |
|  | Rape leaves |  | Other Vegetables | Okra |
| Eggs | Chicken eggs |  |  | Onions |
| Fish &  Seafood | Cichlid (*Utaka*, dried) |  |  | Cabbage |
|  | *Oreochromis lidole*, dry^†^ |  |  | Cucumber |
|  | *Oreochromis lidole*, fresh^†^ |  |  | Eggplant |
|  | Sardine (*Usipa*, sun dried) |  |  | Green beans |
| Flesh Meat | Beef |  | Roots & Tubers | Cassava |
|  | Goat |  |  | Irish potatoes |
|  | Live chicken |  |  | Sweet potatoes |
|  | Pork |  | Salty & fried foods | Mandazi |
| Legumes | Brown beans |  | Sweets &  Confectionary | Biscuits |
|  | Cowpeas |  |  | Brown sugar |
|  | Groundnuts |  |  | White buns |
|  | Pigeon peas |  |  | White sugar |
|  | White beans |  | Stimulants, Spices, &  Condiments^*^ | Salt |
| Milk & Milk Products | Fresh milk |  |  |  |
|  | Powdered milk |  | Caloric beverages^*^ | Coca-cola |
| Oils & Fats | Cooking oil |  |  |  |
|  | Cooking oil refill |  | **Total items (N)** | 51 |

^†^ Tilapia, known locally as *chambo*.

^*^ The food list also monitors the price of three types of tea and a fermented maize-based drink, *Maheu*. Tea is excluded because it confers no essential nutrients. *Maheu* has been excluded from the analysis for lack of food composition data.

Table A-2. Difference between market unit prices and reported unit costs, by size of difference

| **Food item** | **Mean unit cost** | **Mean unit market price** | **Diff. (Market unit price - unit cost)** | **SE (Diff.)** | **p (Diff.)** | | **Degrees of freedom** |
| --- | --- | --- | --- | --- | --- | --- | --- |
| Sun Dried fish (Large Variety) | 2,137 | 5,320 | 3,183.61 | 413.73 | 0.00000 | *** | 43 |
| Sun Dried fish (Medium Variety) | 1,793 | 3,875 | 2,082.35 | 106.28 | 0.00000 | *** | 177 |
| Goat | 1,103 | 3,063 | 1,960.49 | 628.87 | 0.00189 | ** | 753 |
| Beef | 1,178 | 2,787 | 1,608.85 | 789.67 | 0.04232 | * | 374 |
| Sun Dried fish (Small Variety) | 2,015 | 3,150 | 1,134.60 | 44.62 | 0.00000 | *** | 803 |
| Pork | 1,098 | 2,146 | 1,048.04 | 613.15 | 0.08837 |  | 319 |
| Cooking oil | 970 | 1,391 | 421.14 | 16.78 | 0.00000 | *** | 2,282 |
| Maize ufa mgaiwa (normal flour) | 166 | 455 | 288.47 | 6.94 | 0.00000 | *** | 1,092 |
| Fresh milk | 365 | 631 | 266.06 | 137.12 | 0.05321 |  | 326 |
| Maize ufa madeya (bran flour) | 156 | 393 | 237.69 | 30.91 | 0.00000 | *** | 43 |
| Buns, scones | 572 | 747 | 175.23 | 29.98 | 0.00000 | *** | 843 |
| Chicken | 1,326 | 1,490 | 164.43 | 35.63 | 0.00001 | *** | 168 |
| Onion | 328 | 487 | 159.51 | 7.79 | 0.00000 | *** | 2,125 |
| Pigeonpeas | 400 | 553 | 153.42 | 18.35 | 0.00000 | *** | 257 |
| Brown beans | 491 | 610 | 118.84 | 6.10 | 0.00000 | *** | 1,591 |
| Guava | 108 | 223 | 114.45 | 31.49 | 0.00071 | *** | 45 |
| Papaya | 76 | 184 | 107.27 | 19.22 | 0.00001 | *** | 24 |
| Groundnut | 275 | 378 | 103.10 | 8.53 | 0.00000 | *** | 542 |
| Cucumber | 187 | 270 | 82.31 | 32.24 | 0.01458 | * | 40 |
| Avocado | 142 | 217 | 75.62 | 12.29 | 0.00000 | *** | 69 |
| White beans | 424 | 493 | 69.40 | 10.75 | 0.00000 | *** | 472 |
| Tomato | 217 | 284 | 66.91 | 2.89 | 0.00000 | *** | 3,897 |
| Citrus | 122 | 189 | 66.22 | 8.71 | 0.00000 | *** | 144 |
| Cowpeas | 360 | 423 | 63.00 | 13.40 | 0.00000 | *** | 244 |
| Cassava tubers | 90 | 141 | 50.84 | 2.94 | 0.00000 | *** | 688 |
| Rice | 416 | 458 | 42.92 | 3.95 | 0.00000 | *** | 1,052 |
| Pumpkin | 52 | 87 | 34.73 | 7.57 | 0.00005 | *** | 36 |
| White sweet potato | 92 | 113 | 21.35 | 2.02 | 0.00000 | *** | 1,296 |
| Mango | 128 | 142 | 14.24 | 4.93 | 0.00435 | ** | 190 |
| Banana | 152 | 164 | 11.97 | 2.83 | 0.00003 | *** | 1,164 |
| Irish potato | 218 | 213 | -4.74 | 5.60 | 0.39800 |  | 445 |
| Cabbage | 91 | 80 | -10.37 | 1.50 | 0.00000 | *** | 849 |
| Maize grain | 193 | 180 | -13.48 | 22.95 | 0.55971 |  | 49 |
| Chinese cabbage | 230 | 190 | -39.61 | 10.78 | 0.00028 | *** | 348 |
| Mandazi | 708 | 665 | -42.73 | 9.90 | 0.00002 | *** | 1,193 |
| Eggs | 1,071 | 1,026 | -45.88 | 14.88 | 0.00209 | ** | 1,205 |
| Biscuits | 675 | 617 | -57.54 | 63.06 | 0.36230 |  | 277 |
| Rape | 185 | 127 | -57.72 | 2.43 | 0.00000 | *** | 2,844 |
| Pumpkin leaves | 263 | 204 | -59.82 | 5.03 | 0.00000 | *** | 1,228 |
| Okra | 364 | 256 | -108.35 | 9.31 | 0.00000 | *** | 726 |
| Salt | 373 | 232 | -140.63 | 6.80 | 0.00000 | *** | 4,109 |
| Sugar | 671 | 394 | -277.03 | 20.34 | 0.00000 | *** | 2,355 |
| Powdered milk | 7,187 | 1,675 | -5,512.18 | 3,101.76 | 0.07746 |  | 159 |
| Bread | 151,944 | 355 | -151,588.65 | 5,917.13 | 0.00000 | *** | 843 |

Note: Unit costs and unit prices compared in the same month and year in nominal MWK. Extreme unit costs above the 99th percentile excluded. Red font indicates unit costs reported by households exceed market unit prices.

*** p<0.001 ** p<0.01 *p<0.05

Table A-3. Difference between market unit prices and reported unit costs, by food group

|  | **Food item** | **Mean unit cost** | **Mean unit market price** | **Diff. (Market unit price - unit cost)** | **SE (Diff.)** | **p (Diff.)** | | **Degrees of freedom** |
| --- | --- | --- | --- | --- | --- | --- | --- | --- |
| **Staples** | Bread | 151,944 | 355 | -151,588.65 | 5,917.13 | 0.00000 | *** | 843 |
|  | Cassava tubers | 90 | 141 | 50.84 | 2.94 | 0.00000 | *** | 688 |
|  | Irish potato | 218 | 213 | -4.74 | 5.60 | 0.39800 |  | 445 |
|  | Maize grain | 193 | 180 | -13.48 | 22.95 | 0.55971 |  | 49 |
|  | Maize ufa madeya (bran flour) | 156 | 393 | 237.69 | 30.91 | 0.00000 | *** | 43 |
|  | Maize ufa mgaiwa (normal flour) | 166 | 455 | 288.47 | 6.94 | 0.00000 | *** | 1,092 |
|  | Rice | 416 | 458 | 42.92 | 3.95 | 0.00000 | *** | 1,052 |
|  | White sweet potato | 92 | 113 | 21.35 | 2.02 | 0.00000 | *** | 1,296 |
| **Legumes** | Brown beans | 491 | 610 | 118.84 | 6.10 | 0.00000 | *** | 1,591 |
|  | Cowpeas | 360 | 423 | 63.00 | 13.40 | 0.00000 | *** | 244 |
|  | Groundnuts | 275 | 378 | 103.10 | 8.53 | 0.00000 | *** | 542 |
|  | Pigeonpea | 400 | 553 | 153.42 | 18.35 | 0.00000 | *** | 257 |
|  | White beans | 424 | 493 | 69.40 | 10.75 | 0.00000 | *** | 472 |
| **Vegetables** | Cabbage | 91 | 80 | -10.37 | 1.50 | 0.00000 | *** | 849 |
|  | Chinese cabbage | 230 | 190 | -39.61 | 10.78 | 0.00028 | *** | 348 |
|  | Cucumber | 187 | 270 | 82.31 | 32.24 | 0.01458 | * | 40 |
|  | Okra | 364 | 256 | -108.35 | 9.31 | 0.00000 | *** | 726 |
|  | Onion | 328 | 487 | 159.51 | 7.79 | 0.00000 | *** | 2,125 |
|  | Pumpkin | 52 | 87 | 34.73 | 7.57 | 0.00005 | *** | 36 |
|  | Pumpkin leaves | 263 | 204 | -59.82 | 5.03 | 0.00000 | *** | 1,228 |
|  | Rape | 185 | 127 | -57.72 | 2.43 | 0.00000 | *** | 2,844 |
|  | Tomato | 217 | 284 | 66.91 | 2.89 | 0.00000 | *** | 3,897 |
| **Fruits** | Avocado | 142 | 217 | 75.62 | 12.29 | 0.00000 | *** | 69 |
|  | Banana | 152 | 164 | 11.97 | 2.83 | 0.00003 | *** | 1,164 |
|  | Citrus | 122 | 189 | 66.22 | 8.71 | 0.00000 | *** | 144 |
|  | Guava | 108 | 223 | 114.45 | 31.49 | 0.00071 | *** | 45 |
|  | Mango | 128 | 142 | 14.24 | 4.93 | 0.00435 | ** | 190 |
|  | Papaya | 76 | 184 | 107.27 | 19.22 | 0.00001 | *** | 24 |
| **Animal-source foods** | Beef | 1,178 | 2,787 | 1,608.85 | 789.67 | 0.04232 | * | 374 |
|  | Chicken | 1,326 | 1,490 | 164.43 | 35.63 | 0.00001 | *** | 168 |
|  | Eggs | 1,071 | 1,026 | -45.88 | 14.88 | 0.00209 | ** | 1,205 |
|  | Fresh milk | 365 | 631 | 266.06 | 137.12 | 0.05321 |  | 326 |
|  | Goat | 1,103 | 3,063 | 1,960.49 | 628.87 | 0.00189 | ** | 753 |
|  | Pork | 1,098 | 2,146 | 1,048.04 | 613.15 | 0.08837 |  | 319 |
|  | Powdered milk | 7,187 | 1,675 | -5,512.18 | 3,101.76 | 0.07746 |  | 159 |
|  | Sun Dried fish (Large Variety) | 2,137 | 5,320 | 3,183.61 | 413.73 | 0.00000 | *** | 43 |
|  | Sun Dried fish (Medium Variety) | 1,793 | 3,875 | 2,082.35 | 106.28 | 0.00000 | *** | 177 |
|  | Sun Dried fish (Small Variety) | 2,015 | 3,150 | 1,134.60 | 44.62 | 0.00000 | *** | 803 |
|  | Cooking oil | 970 | 1,391 | 421.14 | 16.78 | 0.00000 | *** | 2,282 |
|  | Salt | 373 | 232 | -140.63 | 6.80 | 0.00000 | *** | 4,109 |
| **Sweets** | Biscuits | 675 | 617 | -57.54 | 63.06 | 0.36230 |  | 277 |
|  | Buns, scones | 572 | 747 | 175.23 | 29.98 | 0.00000 | *** | 843 |
|  | Mandazi | 708 | 665 | -42.73 | 9.90 | 0.00002 | *** | 1,193 |
|  | Sugar | 671 | 394 | -277.03 | 20.34 | 0.00000 | *** | 2,355 |

Table A-4. Nutrient Composition and Density by Food Item and Nutrient

| **Nutrient** | **Items with highest nutrient quantity**  **per 100g edible portion*** | **Items with highest nutrient density**  **(quantity per unit energy) *** |
| --- | --- | --- |
| Energy | Cooking oil, Groundnuts, Powdered milk, Biscuits, Sugar, Maize flour, Pigeon peas, Dry *Usipa*, Cowpeas, Rice | Cooking oil, Groundnuts, Powdered milk, Biscuits, Sugar, Maize flour, Pigeon peas, Dried *Usipa*, Cowpeas, Rice grain |
| Carbohydrate | Sugar, Rice, Maize flour. Maize grain, Biscuits, Pigeon peas, Cowpeas, White beans, Brown beans, White bread | Coca-cola, Sugar, Cucumber, Cassava, Mango, Banana, Sweet potato, Oranges, Rice, Papaya |
| Protein | Dry *Chambo*, Dry *Usipa*, *Utaka*, Powdered milk, Brown beans, Groundnuts, Cowpeas, Pigeon peas, White beans, Chicken | Dry *Chambo*, Beef, Dry *Usipa*, Chicken, Fresh *Chambo*, *Utaka*, Goat, Eggs, Pumpkin leaves, Brown beans, Pork |
| Lipids | Cooking oil, Groundnuts, Powdered milk, Pork, Biscuits, *Utaka*, Avocado, Goat, Eggs, Dry *Usipa* | Cooking oil, Avocado, Pork, Groundnuts, Eggs, Goat, Fresh milk, Powdered milk, *Utaka*, Biscuits |
| Vitamin A^†^ | Rape leaves, Powdered milk, Pumpkin, Biscuits, Pumpkin leaves, Mangoes | Rape leaves, Pumpkin leaves, Pumpkin, Chinese cabbage, Mangoes, Tomatoes |
| Retinol | Powdered milk, Chicken, Biscuits, Eggs, Fresh milk | Chicken, Eggs, Fresh milk, Powdered milk, Biscuits |
| Vitamin C | Guava, Papaya, Rape leaves, Oranges, Okra, Chinese cabbage, Cassava, Cabbage, Mangoes, Pumpkin leaves | Guava, Chinese cabbage, Papaya, Rape leaves, Oranges, Cabbage, Pumpkin leaves, Okra, Tomatoes |
| Vitamin E | Cooking oil, Groundnuts | Pumpkin leaves, Rape leaves, Cooking oil, Pumpkin, Tomatoes, Groundnuts, Papaya, Mangoes, Guava |
| Thiamin | Groundnuts, White beans, Pork, Cowpeas, Pigeon peas, Brown beans, White buns, Maize grain, Maize flour | Pork, Irish potatoes, White beans, Cowpeas, White buns, Rape leaves, Green beans, Cucumber, Pumpkin leaves |
| Riboflavin | Powdered milk, Dry *Usipa*, Eggs, Goat, Dry *Chambo*, Brown beans, Pork, White beans, Beef, Pigeon peas | Powdered milk, Eggs, Rape leaves, Pumpkin leaves, Fresh milk, Cucumber, Beef, Okra, Dried *Usipa*, Goat |
| Niacin | Dry *Usipa*, Groundnuts, Beef, Goat, Pork, Chicken, Dry *Chambo*, Cowpeas, Pigeon peas, Maize grain | Dried *Usipa*, Beef, Goat, Chicken, Groundnuts, Chinese cabbage, Tomatoes, Green beans, Irish potatoes, Pumpkin leaves |

| **Nutrient** | **Items with highest nutrient quantity**  **per 100g edible portion^*^** | **Items with highest nutrient density**  **(quantity per unit energy) ^*^** |
| --- | --- | --- |
| Vitamin B6 | Dry *Usipa* | Guava, Dried *Usipa*, Rape leaves, Okra, Pumpkin leaves, Banana, Irish potatoes, Tomatoes, Onions, Cucumber |
| Folate | Cowpeas, Brown beans, White beans, Pigeon peas, Rape leaves, Okra, Groundnuts | Rape leaves, Okra, Cowpeas, Pumpkin leaves, Brown beans, White beans, Pigeon peas |
| Vitamin B12 | Dry *Usipa*, Dry *Chambo*, Eggs, Powdered milk, Beef | Dried *Usipa*, Dry *Chambo*, Beef, Eggs, Goat, Fresh milk, Powdered milk, Pork, Chicken |
| Calcium | Rape leaves, Pumpkin leaves, *Utaka*, Dry *Chambo*, Dry *Usipa*, Cabbage, Papaya, Powdered milk | Pumpkin leaves, Rape leaves, Cabbage, Papaya, Tomatoes, Onions, *Utaka*, Dry *Chambo*, Chinese cabbage, Dried *Usipa* |
| Copper | Tomatoes, Cabbage, Papaya, Sweet potatoes, Pigeon peas, Onions, Pumpkin leaves, Groundnuts, Rape leaves, Cowpeas | Tomatoes, Cabbage, Pumpkin leaves, Papaya, Onions, Rape leaves, Sweet potatoes, Mangoes |
| Iron | Pumpkin leaves, Dry *Chambo*, Cabbage, Rape leaves, Utaka | Pumpkin leaves, Cabbage, Rape leaves, Tomatoes, Dry *Chambo*, Onions, Papaya, Beef, *Utaka* |
| Magnesium | Pumpkin leaves, *Utaka*, Papaya, Rape leaves, Cabbage | Pumpkin leaves, Cabbage, Rape leaves, Papaya, Onions |
| Phosphorus | Dry *Usipa*, Dry *Chambo*, Powdered milk, White beans, Brown beans, Cowpeas, Groundnuts, Pigeon peas, Rice, Maize grain | Dried *Usipa*, Dry *Chambo*, Pumpkin leaves, Beef, Cucumber, Okra, Powdered milk, Fresh milk, White beans, Eggs |
| Selenium | White beans, Pumpkin leaves, Papaya, Brown beans, Tomatoes, Rape leaves, Cabbage, Cowpeas | Pumpkin leaves, Tomatoes, Cabbage, Papaya, Rape leaves, Onions, Mangoes, White beans, Brown beans |
| Zinc | Dry *Usipa*, Rape leaves, Pumpkin leaves, Dry *Chambo*, Onions, Pork, Cabbage, Goat, Powdered milk, Brown beans | Rape leaves, Pumpkin leaves, Tomatoes, Cabbage, Onions, Dried *Usipa*, Chinese cabbage, Papaya, Goat, Beef |
| Sodium | Salt, White bread, White buns, Biscuits, Powdered milk, Dry *Chambo*, Dry *Usipa* | White bread, White buns |

* Listed in descending order of quantity or density. Listing top sources where a natural divide in density or quantity occurs, otherwise top 10 items listed.

^†^ Sugar and cooking oil are fortified with vitamin A in Malawi.

Table A-5. Food Item Median Cost per kg (2011 US$ PPP)

2013 – 2017, Median over All Markets and Months

| **Food Item** | **Cost ($)** |  | **Food Item** | **Cost ($)** |
| --- | --- | --- | --- | --- |
| Maize grain | 1.22 |  | Papaya | 1.15 |
| Maize grain, Admarc^*^ | 0.67 |  | Guava | 1.12 |
| Maize flour (dehulled) | 3.02 |  | Avocado | 1.58 |
| Maize flour (whole grain) | 2.18 |  | Mangoes | 0.84 |
| Rice grain | 3.43 |  | Chicken (live) | 11.44 |
| Irish potatoes | 1.64 |  | Oreochromis lidole, fresh (*Chambo*) | 14.36 |
| Sweet potatoes | 0.84 |  | Oreochromis lidole, dry (*Chambo*) | 31.4 |
| Cassava | 1.17 |  | Cichlid (*Utaka*) | 24.24 |
| White beans | 4.43 |  | Sardine (*Usipa* sun dried) | 17.88 |
| Brown beans | 4.73 |  | Salt | 1.43 |
| Pigeon peas | 3.45 |  | White bread | 2.76 |
| Cowpeas | 3.19 |  | White buns | 5.86 |
| Groundnuts | 5.28 |  | Biscuits | 4.18 |
| Onions | 3.59 |  | Mandazi | 4.29 |
| Tomatoes | 2.03 |  | Beef | 10.13 |
| Cucumber | 1.77 |  | Goat | 9.87 |
| Pumpkin | 0.65 |  | Pork | 8.78 |
| Cabbage | 0.79 |  | Fresh milk | 2.02 |
| Pumpkin leaves | 1.53 |  | Powdered milk^†^ | 17.18 |
| Green beans | 2.9 |  | Eggs | 8.97 |
| Chinese cabbage | 1.35 |  | Cooking oil | 12.92 |
| Okra | 2.2 |  | Cooking oil refill | 6.92 |
| Rape leaves | 1.16 |  | White sugar | 3.85 |
| Eggplant | 1.43 |  | Brown Sugar | 3.9 |
| Banana | 1.77 |  | Coca-cola | 2.35 |
| Oranges | 1.55 |  |  |  |

^*^ Maize grain sold by the parastatal Agricultural Development and Marketing Corporation (Admarc).

^†^ With 1 kg powder yielding approximately 8 liters of liquid milk (1 liter equals 1.01 kg), the median cost per kg liquid added is $2.15.

1. **Policy Scenarios Further Background Information**

In this appendix, we provide additional supporting information used to select and define the policy scenarios. Figure B-1 depicts the energy-adjusted nutrient adequacy ratios, demonstrating the nutrients most under-consumed in current diets. We then proceed with a detailed summary of the context of current availability and prices for all of the food items modeled.

Figure B-1. Nutrient Density Adequacy of Household Diets, 2013 & 2016/17


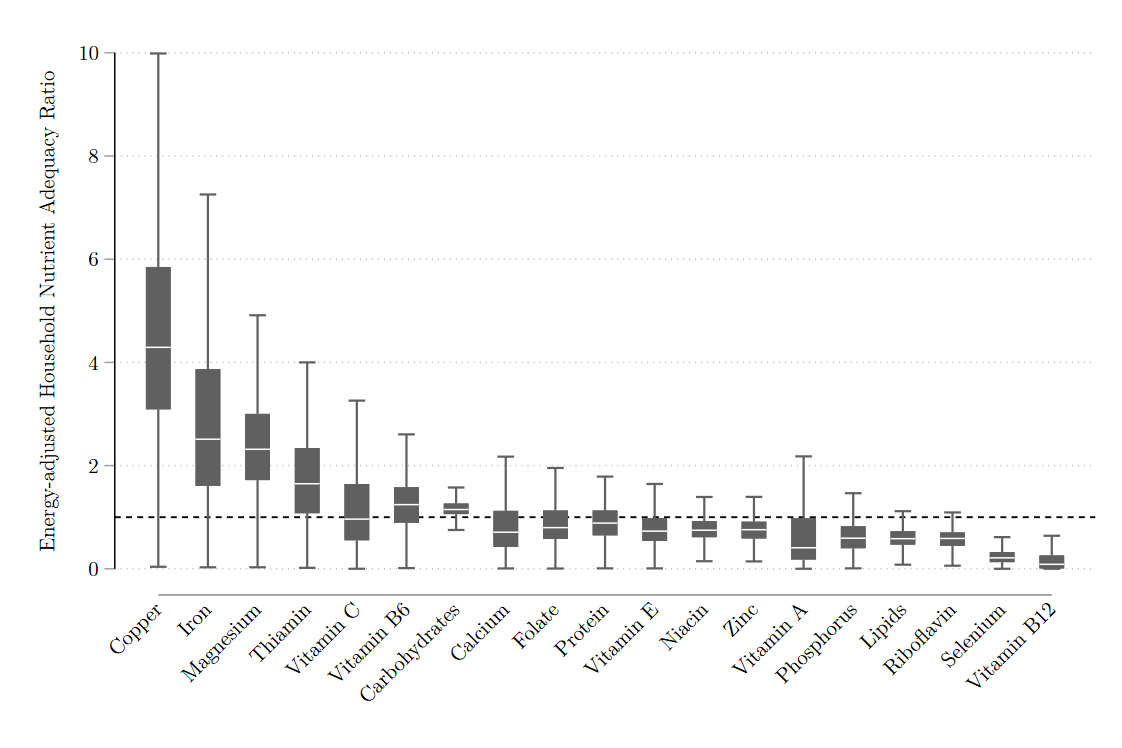


Notes: Black line marks an adequacy ratio of 1 indicating 100% of needs met. Excludes outside values. Adjusted for survey weights.

Eggs are already available in nearly all markets and months at a median price of $5.30 for a crate of 30 eggs (approximately $2.12 per dozen eggs). Only a few markets stand out for lack of availability: Phalombe, Mchinji, and to a lesser extent Nkhatabay and Mitundu (Figure A-2). We chose to model multiple levels of percentage price reduction because eggs often are singled out among animal-source foods by development and nutrition interventions as the (usually) lowest cost, nutrient-dense, and safest item to increase ASFs in the diet (Iannotti, 2018; Iannotti et al., 2019, 2017, 2014; Kim et al., 2019; Lutter et al., 2016; Morris et al., 2018; Omer, 2020; Sen et al., 2017; Stark et al., 2020; Stewart et al., 2019).

Figure B-2. Eggs Availability


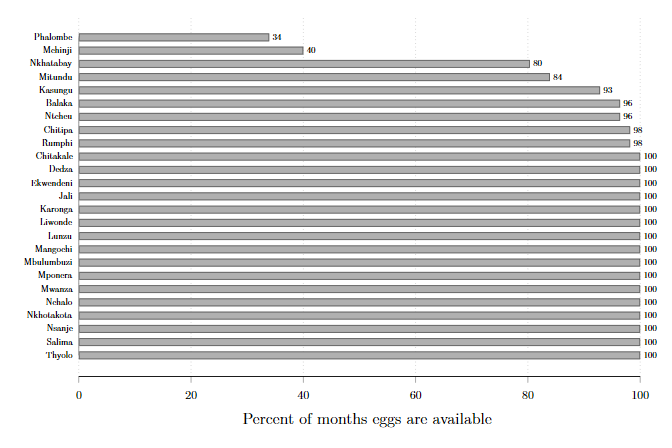


Several varieties of dried fish are monitored in the price dataset. These include very small, dried cichlid (known locally as *utaka*), sardine (known locally as *usipa*), and *oreochromis lidole* (known locally as *chambo*, and commonly as tilapia). Though there is a fishing ban during spawning season in the country’s two largest lakes, the dried fish are storable and could ostensibly be available in all markets and months with adequate storage even under existing biologically- and policy-constrained supply. At present there is a wide range in dried fish availability by market, with eight markets having fish available over 90% of the time while the remainder range from 31-85% (median 69%; Figure A-3). There is large variation in price for each of the three items, with variation not clearly explained by market or season but somewhat by year. Median price per kg is $24.24 for *utaka*, $31.40 for *chambo* and $17.88 for *usipa.* Of note, there is wide variation in the prices of dried fish with standard deviations of the price per kg ranging from $10 to $36.

Figure B-3. Dried Fish Availability


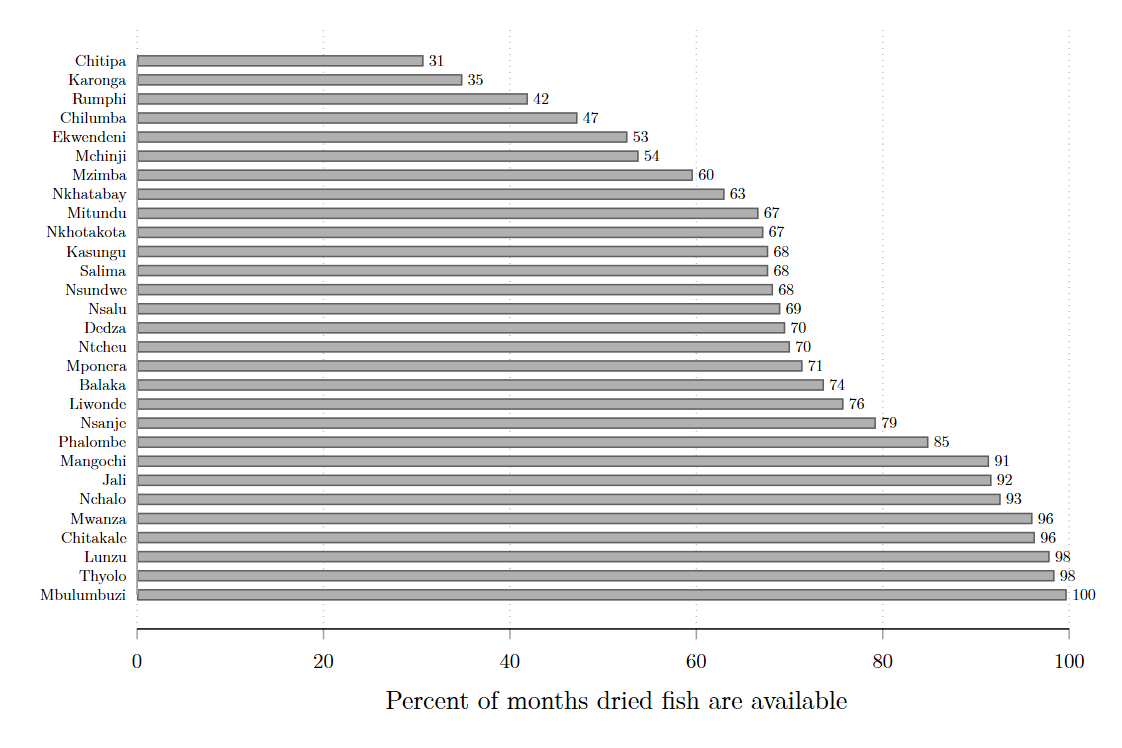


Groundnuts are available in nearly all months for two-thirds of all markets and ranging from 61-86% of months for nine of the ten remaining markets. Nsalu is an outlier with only 26% of months where groundnuts are available (Figure A-4). Median price is $5.28 per kg.

Figure B-4. Groundnuts Availability


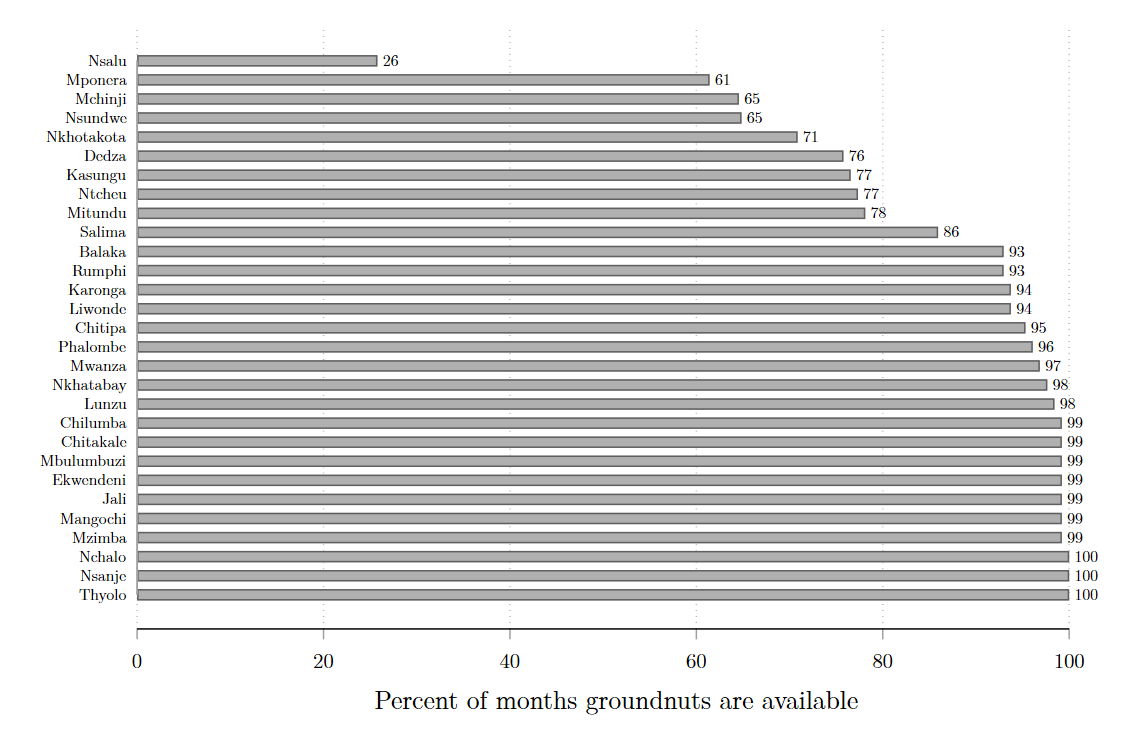


The milk scenarios require a bit more background as powdered milk is a politically sensitive value chain. The government and donors have long supported the development of Malawi’s dairy sector and seen powdered milk as a competitor since there is no domestic production (Kaneene et al., 2016). However, from a nutritional perspective, fresh and powdered milk are nutritionally equivalent products with alternative value chains and potential policy approaches. Fresh milk is available in most markets in almost all months at a median price of $2.02/kg (approximately $2.08/liter) (Figure A-5). A small proportion of markets have more limited availability (Jali, Mangochi, Phalombe, Balaka, Ntcheu, Mitundu). However, the fresh milk value chain suffers from a number of on-farm inefficiencies limiting production and productivity (e.g. animal distribution through a donor-driven pass-on scheme focused on pure exotic breeds, feed shortage, lack of access to animal health services), concentration of market power for pasteurization, and a large proportion sold unsafely on the informal market. Therefore numerous challenges face the sector and improving safe supply substantially enough to lower consumer prices would require a number of coordinated actions along the value chain (Baur et al., 2017; Revoredo-Giha, 2019).

Powdered milk has the triple advantages that it is non-perishable, poses low food safety risk if mixed with clean water, and in being centrally processed can be fortified with additional nutrients. At present there has been a wide range in the frequency of availability of fresh and powdered milk over the January 2013–July 2017 period (Figure A-6). Powdered milk is always available in a few markets (Thyolo, Lunzu, Mwanza, Nchalo) but also never available in a few others (Nsanje, Mangochi, Jali; the latter two of these have limited fresh milk as well). On average, it is available 60% of the time over all markets and months at a median price of $17.18/kg (2011 US$ PPP) in the concentrated powdered form, with one kg powder yielding approximately eight liters of liquid milk the median price is equivalent to $2.14/liter, nearly the same as that for fresh milk. The current trade policy imposes no barriers at the border, though stakeholders in the domestic dairy sector have lobbied for such.^^[[1]](#footnote-1)^^

Figure B-5. Fresh Milk Availability


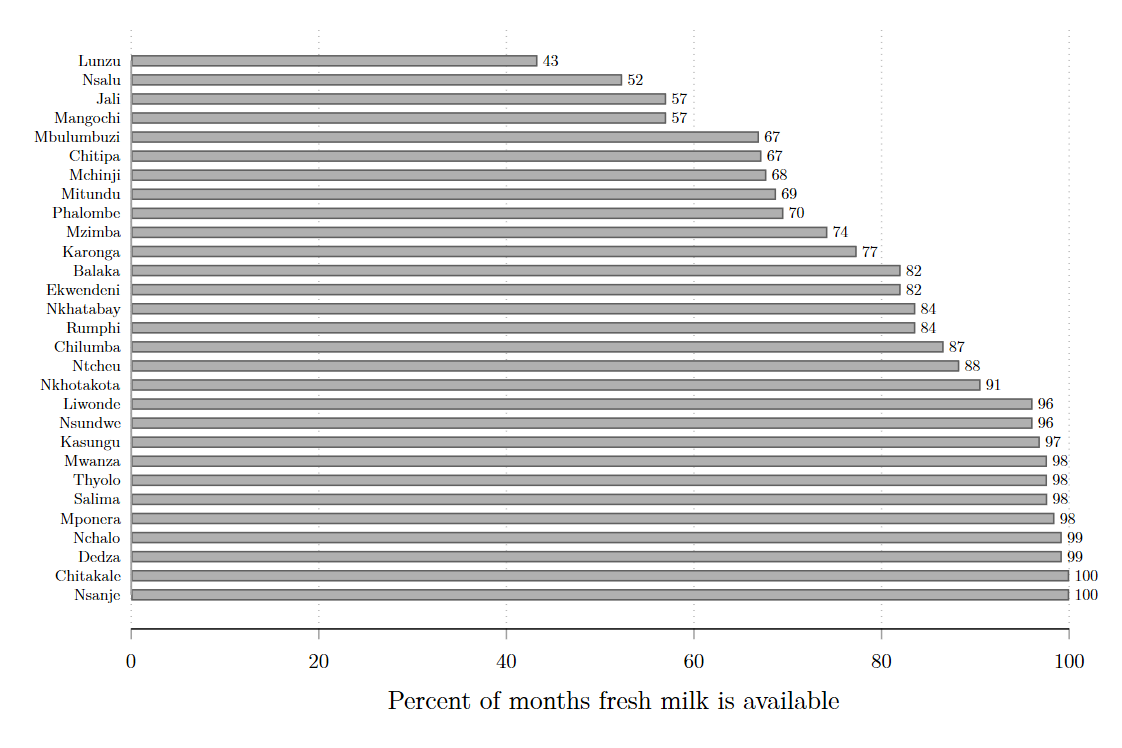


Figure B-6. Powdered Milk Availability


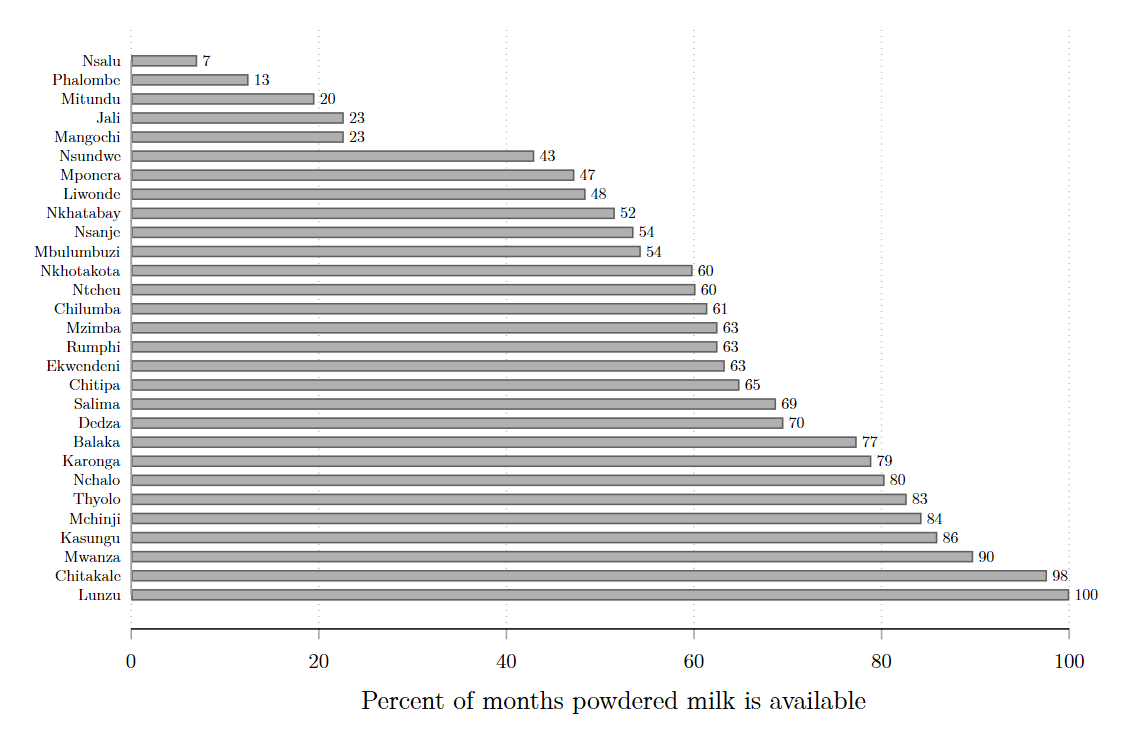


The final scenario we model is soil biofortification with selenium. The selenium content of plants is largely determined by soil mineral content, and both soil minerals and household diets have been shown to be seriously deficient in selenium, consistent with our findings in prior research (Chapter 4 and Figure B-1, this appendix) (Hurst et al., 2013; Joy et al., 2015; Ligowe et al., 2020a; Phiri et al., 2019). Field trials were carried out over the last ten years to determine the optimal dosing that results in desirable selenium content in the edible maize grain. The trials were designed to achieve optimal fortification composition whereby needs are met for most of the population while risk of excess intakes given current levels of consumption are low (Chilimba, 2011; Chilimba et al., 2014, 2012a, 2012b; Joy et al., 2019; Ligowe et al., 2020b).

Emerging results from the human nutrition trial of the consumption of soil-biofortified maize has shown it to be effective and safe in increasing women’s selenium status (Joy, 2020a, 2020b; Joy et al., 2019). Based on those studies and confirmation with the researchers, we model a scenario where the selenium composition of whole maize and whole grain flour are 11.3 mcg per 100 g edible portion and 5.3 mcg per 100 g edible portion for dehulled maize flour (ufa woyera) (Joy, 2020a). For this scenario, we make no changes to availability or prices. Maize is considered the primary food security crop in Malawi and is universally available in at least some form; maize flour is available in 99% of all market-months while whole grain maize is present 80% of the time. Furthermore, Malawi already has a large input subsidy program including fertilizer and maize seed, and therefore the potential to augment the nutrient blend with selenium through the existing production and distribution system is plausible.

We considered modeling the obvious case of universal compliance with existing fortification law, but determined it was not among the most feasible options or possible to model for several reasons. Current policy since 2011 mandates the fortification of all maize and wheat flour as well as rice to be fortified with micronutrients. However, only 15% of maize flour and 0% of rice are industrially processed and the opportunity for fortification is considered low by the Global Fortification Data Exchange (2020). The most realistic compliance scenario for the fortification law would be that all wheat flour and processed products made from wheat adhere to the fortification policy. The policy requires the following micronutrient composition per kilogram of wheat flour: 30 mg iron, 2 mg folate, 50 mg niacin, 80 mg zinc, 6 mg riboflavin, 9 mg thiamin, 0.02 mg vitamin B12, one mg vitamin A (Global Fortification Data Exchange, 2020b; Malawi Bureau of Standards, 2017).

Straight wheat flour is not included in the list of food items for which prices are collected, meaning households spent less than 0.02% of total expenditure on wheat flour in 2010. There are four items made from wheat flour: white bread, white buns, mandazi (fried dough) and biscuits. Though white buns and mandazi are generally made at the local level, the wheat flour from which they are made would almost certainly be centrally processed. As of September 2018, the Ministry of Health reported 20% of wheat flour – based on household and market samples – was fortified in compliance with the law. White bread is centrally processed, however given low reported compliance with the wheat fortification law we suspect the samples used for the composition analysis were unlikely to reflect a high level of compliance with the law. We do acknowledge fortification could occur at the bread factory and would not be reflected in the statistics regarding compliance in wheat flour and therefore remain uncertain about whether the food composition data already reflect fortification or not. No Malawi-specific food composition data are available for biscuits and therefore we use a generic unenriched butter cookie from USDA data and therefore our results might be sensitive to this choice.

1. **Household Composition**

Figure C-1. Diet Feasibility and Cost by Household Composition


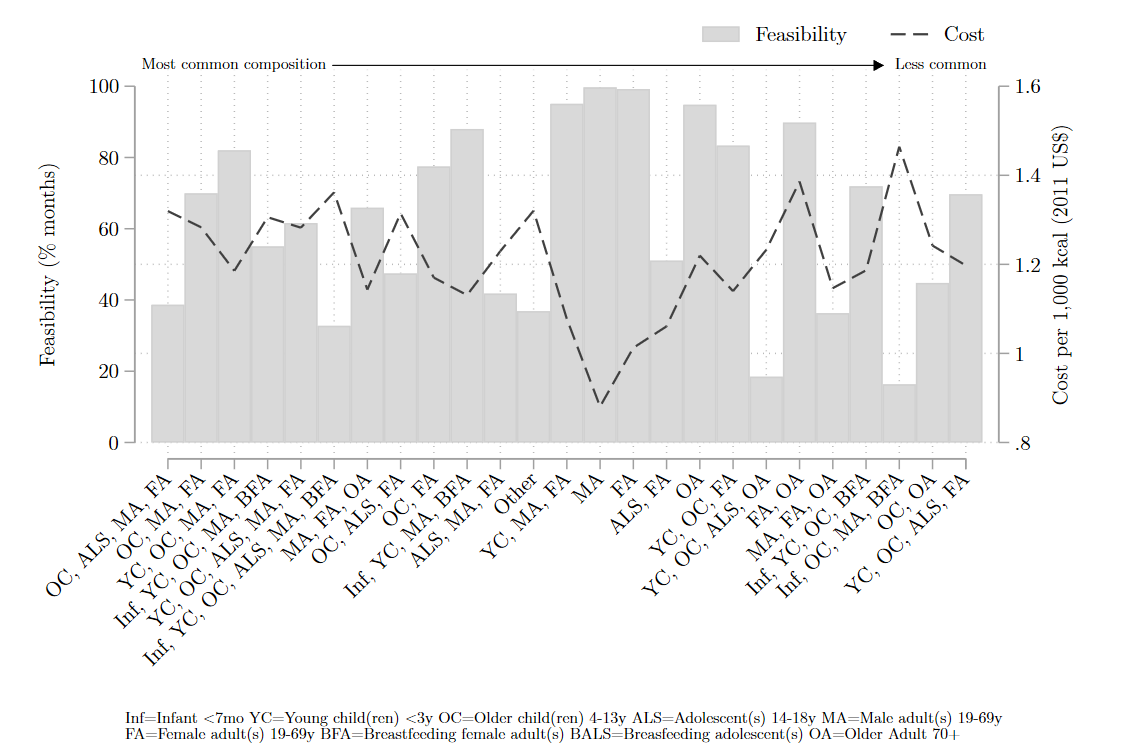


Population statistics calculated using sampling weights. Outliers (defined as households with a HHCoNA more extreme than 1.5 times the IQR) excluded. Compositions observed in <1% of all households in the population not shown. “Other” accounts for 2.5% of the total population but contains compositions observed in fewer than five households each.

Table C-1 summarizes the average nutrient requirements at the population level with person-level survey data, presenting survey weighted averages over the entire population. Column 1 reflects average lower and upper bound nutrient requirements as defined by the DRIs. Column 2 reflects the lower and upper bounds under the shared household diet. Column 3 shows the percentage difference between Column 1 and Column 2.

Table C-1. Household Composition Frequencies

| ***Composition*** |  | Households (%) |  | Feasibility (%) | |  | Cost per 1,000 kcal  (2011 US$) | |
| --- | --- | --- | --- | --- | --- | --- | --- | --- |
|  |  |  |  | Mean | (SE) |  | Mean | (SE) |
| Older kid(s), adolescent(s), male and female adults |  | 16.8 |  | 38.67 | (2.78) |  | 1.32 | (0.02) |
| Older kid(s), male and female adults |  | 12.1 |  | 69.93 | (2.61) |  | 1.28 | (0.02) |
| Young kid(s), older kid(s), male and female adults |  | 7.8 |  | 82.02 | (2.36) |  | 1.19 | (0.02) |
| Young kid(s), older kid(s), male and breastfeeding female adults |  | 6.8 |  | 55.04 | (2.65) |  | 1.31 | (0.02) |
| Young kid(s), older kid(s), adolescent(s), male and female adults |  | 5.9 |  | 61.58 | (2.10) |  | 1.28 | (0.02) |
| Young kid(s), older kid(s), adolescent(s), male and breastfeeding female adults |  | 4.4 |  | 32.75 | (3.17) |  | 1.36 | (0.02) |
| Male and female adults |  | 3.9 |  | 77.47 | (4.12) |  | 1.17 | (0.06) |
| Older kid(s), adolescent(s), adult female(s) |  | 3.6 |  | 65.89 | (4.98) |  | 1.14 | (0.02) |
| Older kid(s), adult female(s) |  | 3.4 |  | 47.45 | (5.86) |  | 1.31 | (0.04) |
| Young kid(s), male and breastfeeding female adults |  | 2.7 |  | 88.00 | (1.93) |  | 1.13 | (0.03) |
| Adolescent(s), male and female adults |  | 2.6 |  | 36.87 | (5.10) |  | 1.32 | (0.04) |
| Other |  | 2.5 |  | 41.80 | (4.36) |  | 1.23 | (0.04) |
| Young kid(s), male and female adults |  | 2.4 |  | 99.69 | (0.17) |  | 0.88 | (0.03) |
| Adult male(s) |  | 2.3 |  | 95.07 | (1.09) |  | 1.08 | (0.03) |
| Adult female(s) |  | 2.0 |  | 99.21 | (0.38) |  | 1.01 | (0.05) |
| Adolescent(s), female adult(s) |  | 1.9 |  | 51.05 | (7.13) |  | 1.06 | (0.04) |
| Older adult(s) |  | 1.5 |  | 83.35 | (3.15) |  | 1.14 | (0.04) |
| Young kid(s), older kid(s), adult female(s) |  | 1.5 |  | 94.80 | (1.38) |  | 1.22 | (0.05) |
| Young kid(s), older kid(s), adolescent(s), older adult(s) |  | 1.1 |  | 89.79 | (2.10) |  | 1.39 | (0.04) |
| Adult female(s), older adult(s) |  | 1.1 |  | 18.47 | (4.93) |  | 1.23 | (0.05) |
| Working age couple, older adult(s) |  | 1.0 |  | 71.91 | (5.39) |  | 1.19 | (0.03) |
| Young kid(s), older kid(s), adult female(s), breastfeeding |  | 1.0 |  | 16.34 | (4.90) |  | 1.46 | (0.04) |
| Infant, older kid(s), male and breastfeeding female adults |  | 1.0 |  | 36.31 | (4.71) |  | 1.15 | (0.06) |
| Older kid(s), older adult(s) |  | 1.0 |  | 44.78 | (11.26) |  | 1.24 | (0.04) |
| Young kid(s), older kid(s), adolescent(s), adult female(s) |  | 1.0 |  | 69.69 | (4.53) |  | 1.20 | (0.04) |

| ***Composition*** |  | Households (%) |  | Feasibility (%) | |  | Cost per 1,000 kcal  (2011 US$) | |
| --- | --- | --- | --- | --- | --- | --- | --- | --- |
|  |  |  |  | Mean | (SE) |  | Mean | (SE) |
| Adolescent(s), adult female(s), older adult(s) |  | 0.8 |  | 47.63 | (8.32) |  | 1.23 | (0.05) |
| Older kid(s), adolescent(s), older adult(s) |  | 0.7 |  | 38.10 | (15.82) |  | 1.11 | (0.11) |
| Older kid(s), adult female(s), older adult(s) |  | 0.6 |  | 16.79 | (6.41) |  | 1.09 | (0.03) |
| Older kid(s), adolescent(s), male and female adults, older adult(s) |  | 0.6 |  | 39.50 | (9.01) |  | 1.27 | (0.04) |
| Adolescent(s), male and female adults, older adult(s) |  | 0.6 |  | 7.27 | (3.89) |  | 1.54 | (0.12) |
| Adolescent(s), older adult(s) |  | 0.5 |  | 40.31 | (6.91) |  | 1.09 | (0.04) |
| Young kid(s), older kid(s), adolescent(s), adult female(s), breastfeeding |  | 0.5 |  | 55.73 | (5.42) |  | 1.31 | (0.04) |
| Infant, male, and breastfeeding female adults |  | 0.5 |  | 3.48 | (3.19) |  | 1.33 | (0.02) |
| Older kid(s), adolescent(s), adult male(s) |  | 0.5 |  | 63.18 | (11.08) |  | 1.21 | (0.07) |
| Adolescent(s), male and female adults, older adult(s) |  | 0.4 |  | 77.25 | (11.11) |  | 1.21 | (0.05) |
| Young kid(s), adolescent(s), male and female adults |  | 0.4 |  | 90.41 | (4.05) |  | 1.02 | (0.07) |
| Young kid(s), adult female(s) |  | 0.4 |  | 14.27 | (4.86) |  | 1.26 | (0.08) |
| Older kid(s), adolescent(s), male and breastfeeding female adults |  | 0.3 |  | 3.72 | (3.64) |  | 1.57 | (.) |
| Young kid(s), adult female(s), breastfeeding |  | 0.3 |  | 78.87 | (5.75) |  | 1.11 | (0.04) |
| Young kid(s), older kid(s), adolescent(s), male and female adults, older adult(s), breastfeeding |  | 0.3 |  | 44.16 | (10.92) |  | 1.96 | (0.05) |
| Young kid(s), older kid(s), male and female adults, older adult(s), breastfeeding |  | 0.3 |  | 18.54 | (7.93) |  | 1.28 | (0.03) |
| Older kid(s), adult male(s) |  | 0.2 |  | 53.97 | (7.40) |  | 1.00 | (0.02) |
| Adolescent(s), adult male(s) |  | 0.2 |  | 85.66 | (3.18) |  | 1.33 | (0.07) |
| Young kid(s), adolescent(s), breastfeeding |  | 0.2 |  | 72.04 | (10.36) |  | 1.13 | (0.04) |
| Young kid(s), adolescent(s), male and breastfeeding female adults |  | 0.2 |  | 17.03 | (4.54) |  | 1.14 | (0.14) |
| Adult male(s), older adult(s) |  | 0.2 |  | 80.47 | (9.14) |  | 1.00 | (0.09) |
| Young kid(s), older kid(s), male and female adults, older adult(s) |  | 0.1 |  | 37.61 | (18.13) |  | 1.47 | (0.08) |
| Young kid(s), adolescent(s), adult male(s) |  | 0.1 |  | 36.80 | (7.73) |  | 1.28 | (0.04) |
| Adolescent(s), breastfeeding |  | 0.1 |  | 100.00 | (.) |  | 0.89 | (0.02) |
| Adolescent(s) |  | 0.1 |  | 86.27 | (11.53) |  | 0.74 | (0.16) |

Population statistics calculated using sampling weights. Composition types sorted by frequency observed.

Definition of age groups aggregates the age groups in the DRIs as follows: Young = 3 and below, Older kids = 4-13, Adolescent = 14-18, Adult = 19-69, Older adult = 70 and above.

1. **Supplementary Results**

Table D-1 summarizes nutrient requirements for Malawi’s rural population (individual level data), using survey weights to obtain nationally representative averages. Column 1 reflects the lower and upper bounds that would apply for individual diets, while column 2 shows the lower and upper bounds that result for individuals when eating the diet shared with other members of their household. Column 3 shows the difference between 1 and 2, in percentage terms. Results are expressed as the population-weighted average needs per 1,000 calories defined by the DRIs, with household sharing, and the percent difference between the two that corresponds to what is illustrated in Figure 1.

Table D-1. Nutrient Requirements and Upper Limits Per 1,000 kcal

|  | (1) | |  | (2) | |  | (3) | |
| --- | --- | --- | --- | --- | --- | --- | --- | --- |
|  | Individual  (DRIs) | |  | Household  Sharing | |  | % Diff | |
|  | Mean | (SE) |  | Mean | (SE) |  | Mean | (SE) |
| ***Panel A: Lower Bounds*** |  |  |  |  |  |  |  |  |
| Carbohydrate^*^ (g) | 112.50 | (0.00) |  | 113.15 | (0.03) |  | 0.6 | (0.03) |
| Protein (g) | 24.03 | (0.04) |  | 25.07 | (0.00) |  | 8.2 | (0.33) |
| Lipids (g) | 24.87 | (0.05) |  | 29.13 | (0.11) |  | 18.7 | (0.32) |
| Vitamin A (mcg) | 231.30 | (0.49) |  | 288.89 | (1.44) |  | 29.0 | (0.71) |
| Vitamin C (mg) | 23.99 | (0.11) |  | 33.64 | (0.14) |  | 61.5 | (1.36) |
| Vitamin E (mg) | 5.12 | (0.01) |  | 6.43 | (0.01) |  | 31.4 | (0.29) |
| Thiamin (mg) | 0.40 | (0.00) |  | 0.48 | (0.00) |  | 23.6 | (0.25) |
| Riboflavin (mg) | 0.42 | (0.00) |  | 0.50 | (0.00) |  | 21.2 | (0.32) |
| Niacin (mg) | 4.90 | (0.01) |  | 5.79 | (0.01) |  | 22.0 | (0.22) |
| Vitamin B6 (mg) | 0.47 | (0.00) |  | 0.63 | (0.00) |  | 43.2 | (0.71) |
| Folate (mcg) | 135.76 | (0.33) |  | 174.02 | (0.35) |  | 33.4 | (0.35) |
| Vitamin B12 (mcg) | 0.82 | (0.00) |  | 1.04 | (0.00) |  | 32.8 | (0.29) |
| Calcium (mg) | 463.87 | (1.80) |  | 606.22 | (2.23) |  | 41.2 | (0.56) |
| Copper (mg) | 0.29 | (0.00) |  | 0.38 | (0.00) |  | 35.2 | (0.41) |
| Iron (mg) | 3.31 | (0.02) |  | 7.09 | (0.02) |  | 143.6 | (0.75) |
| Magnesium (mg) | 112.05 | (0.49) |  | 142.20 | (0.38) |  | 36.3 | (0.61) |
| Phosphorus (mg) | 352.05 | (1.55) |  | 527.48 | (3.55) |  | 67.9 | (1.04) |
| Selenium (mcg) | 18.75 | (0.04) |  | 23.88 | (0.05) |  | 32.7 | (0.32) |
| Zinc (mg) | 3.42 | (0.01) |  | 6.46 | (0.02) |  | 96.0 | (0.65) |

^*^ Carbohydrate range does not change under household sharing, slight differences due to rounding.

|  | (1) | |  | (2) | |  | (3) | |
| --- | --- | --- | --- | --- | --- | --- | --- | --- |
|  | Individual  (DRIs) | |  | Household  Sharing | |  | % Diff | |
|  | Mean | (SE) |  | Mean | (SE) |  | Mean | (SE) |
| ***Panel B: Upper Bounds^†^*** |  |  |  |  |  |  |  |  |
| Carbohydrate^*^ (g) | 162.50 | (0.00) |  | 163.15 | (0.03) |  | 0.41 | (0.02) |
| Protein (g) | 78.68 | (0.16) |  | 68.03 | (0.43) |  | -12.37 | (0.39) |
| Lipids (g) | 39.32 | (0.02) |  | 39.08 | (0.01) |  | -0.49 | (0.04) |
| Retinol (mcg) | 1083.87 | (5.34) |  | 673.91 | (6.94) |  | -31.83 | (0.30) |
| Vitamin C (mg) | 721.37 | (3.58) |  | 463.75 | (4.70) |  | -30.74 | (0.32) |
| Vitamin B6 (mg) | 37.29 | (0.15) |  | 27.33 | (0.14) |  | -22.57 | (0.15) |
| Calcium (mg) | 1486.71 | (10.03) |  | 917.15 | (4.10) |  | -28.93 | (0.32) |
| Copper (mg) | 3.30 | (0.02) |  | 1.82 | (0.03) |  | -37.85 | (0.52) |
| Iron (mg) | 24.98 | (0.20) |  | 16.57 | (0.07) |  | -23.89 | (0.28) |
| Phosphorus (mg) | 1997.10 | (6.45) |  | 1435.39 | (5.42) |  | -23.24 | (0.26) |
| Selenium (mcg) | 154.45 | (0.60) |  | 104.30 | (0.95) |  | -28.31 | (0.34) |
| Zinc (mg) | 14.08 | (0.07) |  | 8.64 | (0.10) |  | -33.06 | (0.35) |
| Sodium (mg) | 1037.13 | (2.22) |  | 811.27 | (2.59) |  | -18.66 | (0.18) |

Population statistics calculated using sampling weights. Heteroskedasticity robust standard errors in parentheses.

***^†^*** Only relevant nutrients shown, excluded nutrients have no upper bound.

^*^ Carbohydrate range does not change under household sharing, slight differences due to rounding.

**References**

Baur, I., Tabin, L., Banda, M.L., Chiumia, D., Lips, M., 2017. Improving dairy production in Malawi: a literature review. Trop. Anim. Health Prod. 49, 251–258. https://doi.org/10.1007/s11250-016-1184-5

Chilimba, A.D.C., 2011. Potential for safe and efficient biofortification of maize crops with selenium. University of Nottingham.

Chilimba, A.D.C., Young, S.D., Black, C.R., Meacham, M.C., Lammel, J., Broadley, M.R., 2012a. Assessing residual availability of selenium applied to maize crops in Malawi. F. Crop. Res. 134, 11–18. https://doi.org/10.1016/j.fcr.2012.04.010

Chilimba, A.D.C., Young, S.D., Black, C.R., Meacham, M.C., Lammel, J., Broadley, M.R., 2012b. Agronomic biofortification of maize with selenium (Se) in Malawi. F. Crop. Res. 125, 118–128. https://doi.org/10.1016/j.fcr.2011.08.014

Chilimba, A.D.C., Young, S.D., Joy, E.J.M., 2014. Agronomic biofortification of maize, soybean and groundnut with selenium in intercropping and sole cropping systems. African J. Agric. Res. 9, 3620–3626. https://doi.org/10.5897/AJAR2014.8978

Global Fortification Data Exchange, 2020a. Quantity and Proportion of Food Vehicle that is Fortified - Malawi [WWW Document]. URL https://fortificationdata.org/map-proportion-of-food-vehicle-that-is-fortified/ (accessed 7.7.20).

Global Fortification Data Exchange, 2020b. Dashboard: Malawi Fortification [WWW Document]. URL https://fortificationdata.org/country-fortification-dashboard/ (accessed 7.7.20).

Hurst, R., Siyame, E.W.P., Young, S.D., Chilimba, A.D.C., Joy, E.J.M., Black, C.R., Ander, E.L., Watts, M.J., Chilima, B., Gondwe, J., Kang’Ombe, D., Stein, A.J., Fairweather-Tait, S.J., Gibson, R.S., Kalimbira, A.A., Broadley, M.R., 2013. Soil-type influences human selenium status and underlies widespread selenium deficiency risks in Malawi. Sci. Rep. 3. https://doi.org/10.1038/srep01425

Iannotti, L.L., 2018. The benefits of animal products for child nutrition in developing countries. Rev. Sci. Tech. 37, 37–46. https://doi.org/10.20506/rst.37.1.2738

Iannotti, L.L., Chapnick, M., Nicholas, J., Gallegos-Riofrío, C.A., Moreno, P., Douglas, K., Habif, D., Cui, Y., Stewart, C.P., Lutter, C.K., Waters, W.F., 2019. Egg intervention effect on linear growth no longer present after two years. Matern. Child Nutr. 1–10. https://doi.org/10.1111/mcn.12925

Iannotti, L.L., Lutter, C.K., Bunn, D.A., Stewart, C.P., 2014. Eggs: the uncracked potential for improving maternal and young child nutrition among the world’s poor. Nutr. Rev. 72, 355–368. https://doi.org/10.1111/nure.12107

Iannotti, L.L., Lutter, C.K., Stewart, C.P., Riofrío, C.A.G., Malo, C., Reinhart, G., Palacios, A., Karp, C., Chapnick, M., Cox, K., Waters, W.F., 2017. Eggs in early complementary feeding and child growth: A randomized controlled trial. Pediatrics 140. https://doi.org/10.1542/peds.2016-3459

Joy, E.J.M., 2020a. Personal Communication.

Joy, E.J.M., 2020b. Primary Findings of the Addressing Hidden Hunger with Agronomy (AHHA) Trial, in: ANH 2020: Agriculture, Nutrition and Health Academy Week. Virtual.

Joy, E.J.M., Broadley, M.R., Watts, M.J., Chilimba, A.D.C., Young, S., Ander, L., Black, C.R., 2015. Estimates of Dietary Mineral Supply in Malawi Based on Soil Type and Household Survey Data. Eur. J. Nutr. Food Saf. 5, 642–643. https://doi.org/10.9734/EJNFS/2015/21007

Joy, E.J.M., Kalimbira, A.A., Gashu, D., Ferguson, E.L., Sturgess, J., Dangour, A.D., Banda, L., Chiutsi-Phiri, G., Bailey, E.H., Langley-Evans, S.C., Lark, R.M., Millar, K., Young, S.D., Matandika, L., Mfutso-Bengo, J., Phuka, J.C., Phiri, F.P., Gondwe, J., Ander, E.L., Lowe, N.M., Nalivata, P.C., Broadley, M.R., Allen, E., 2019. Can selenium deficiency in Malawi be alleviated through consumption of agro-biofortified maize flour? Study protocol for a randomised, double-blind, controlled trial. Trials 20, 795. https://doi.org/10.1186/s13063-019-3894-2

Kaneene, J.B., Thiagarajan, D., Chigwa, F., Gondwe, T., Gunaseelan, L., Thirunavukkarasu, M., Babu, M., Balakrishnan, V., Kambewa, D., Mvula, R., Dzanja, J., Bakili, O., Mlotha, V., Kakwera, M., Chmombo, M., Mwangela, A., Sivaselvam, S.N., Miller, R., Asokan, S.A., Palanidorai, R., Prathaban, S., Kumanan, K., Ramesh Saravanakumar, R., Mohan Kumar, P.S., 2016. A tri-lateral capacity building approach to strengthen the dairy value chain in Malawi: Overview of the design and implementation. Livest. Res. Rural Dev. 28.

Kim, S.S., Nguyen, P.H., Yohannes, Y., Abebe, Y., Tharaney, M., Drummond, E., Frongillo, E.A., Ruel, M.T., Menon, P., 2019. Behavior change interventions delivered through interpersonal communication, agricultural activities, community mobilization, and mass media increase complementary feeding practices and reduce child stunting in Ethiopia. J. Nutr. 149, 1470–1481. https://doi.org/10.1093/jn/nxz087

Ligowe, I.S., Phiri, F.P., Ander, E.L., Bailey, E.H., Chilimba, A.D.C., Gashu, D., Joy, E.J.M., Lark, R.M., Kabambe, V., Kalimbira, A.A., Kumssa, D.B., Nalivata, P.C., Young, S.D., Broadley, M.R., 2020a. Selenium deficiency risks in sub-Saharan African food systems and their geospatial linkages, in: Proceedings of the Nutrition Society. Cambridge University Press, pp. 1–11. https://doi.org/10.1017/S0029665120006904

Ligowe, I.S., Young, S.D., Ander, E.L., Kabambe, V., Chilimba, A.D.C., Bailey, E.H., Lark, R.M., Nalivata, P.C., 2020b. Selenium biofortification of crops on a Malawi Alfisol under conservation agriculture. Geoderma 369. https://doi.org/10.1016/j.geoderma.2020.114315

Lutter, C.K., Iannotti, L.L., Stewart, C.P., 2016. Cracking the Egg Potential During Pregnancy and Lactation. Sight Life 75–81.

MAFOODS, 2019. Malawian Food Composition Table, 1st ed. Lilongwe, Malawi.

Malawi Bureau of Standards, 2017. Catalogue of Malawi Standards. Malawi Bureau of Standards, Blantyre, Malawi.

Morris, S.S., Beesabathuni, K., Headey, D.D., 2018. An egg for everyone: Pathways to universal access to one of nature’s most nutritious foods. Matern. Child Nutr. 14, 1–9. https://doi.org/10.1111/mcn.12679

Omer, A., 2020. Poultry interventions and child nutrition status in low-income countries. African J. Food, Agric. Nutr. Dev. 20, 16013–16028. https://doi.org/10.18697/ajfand.92.19105

Phiri, F.P., Ander, E.L., Bailey, E.H., Chilima, B., Chilimba, A.D.C., Gondwe, J., Joy, E.J.M., Kalimbira, A.A., Kumssa, D.B., Lark, R.M., Phuka, J.C., Salter, A., Suchdev, P.S., Watts, M.J., Young, S.D., Broadley, M.R., 2019. The risk of selenium deficiency in Malawi is large and varies over multiple spatial scales. Sci. Rep. 9, 1–8. https://doi.org/10.1038/s41598-019-43013-z

Revoredo-Giha, C., 2019. An analysis of the tax incidence of VAT to milk in Malawi, in: 6th African Conference of Agricultural Economists. Abuja, Nigeria.

Sen, P., Mardinogulu, A., Nielsen, J., 2017. Selection of complementary foods based on optimal nutritional values. Sci. Rep. 7, 5413. https://doi.org/10.1038/s41598-017-05650-0

Stark, H., Omer, A., Wereme N’Diaye, A., Sapp, A.C., Moore, E. V., McKune, S.L., 2020. The Un Oeuf study: Design, methods and baseline data from a cluster randomised controlled trial to increase child egg consumption in Burkina Faso. Matern. Child Nutr. https://doi.org/10.1111/mcn.13069

Stewart, C.P., Caswell, B., Iannotti, L.L., Lutter, C.K., Arnold, C.D., Chipatala, R., Prado, E.L., Maleta, K.M., 2019. The effect of eggs on early child growth in rural Malawi: The Mazira Project randomized controlled trial. Am. J. Clin. Nutr. 110, 1026–1033. https://doi.org/10.1093/ajcn/nqz163

United Nations Department of Economic and Social Affairs, S.D., n.d. UN Comtrade: International Trade Statistics [WWW Document]. URL https://comtrade.un.org/data (accessed 7.7.20).

1. Main trading partners from 2014 – 2017 were Ireland (accounting for nearly half of all powdered milk imports by quantity), the UK (25%), Malaysia (21%), Belgium (17%), South Africa (14%), the Netherlands (13%), Sweden (13%), and Singapore (10%) (United Nations Department of Economic and Social Affairs). Food composition data reflects South African food composition (MAFOODS, 2019). [↑](#footnote-ref-1)
